# Supplementary material for: Exploring the impact of mobility and selection on stone tool recycling behaviors through agent-based simulation
Source: PLoS One. 2023 Nov 9;18(11):e0294242. doi: 10.1371/journal.pone.0294242 (PMC10635449; doi:10.1371/journal.pone.0294242)
Supplement: S1 Text — Description of the recycling agent-based model used in this paper following the ODD protocol. (DOCX) [file pone.0294242.s001.docx]

**Exploring the impact of mobility and selection on recycling behaviors through agent-based simulation**

Emily Coco

Corresponding author: Emily Coco – Center for Study of Human Origins, NYU

Corresponding email: ec3307@nyu.edu

**Overview, Design Concepts, and Details for extended recycling model**

The following description of the recycling agent-based model follows the ODD protocol for describing individual- and agent-based models (Grimm et al. 2006, 2010, 2020).

**Purpose**

The main purpose of this model is to understand how recycling behaviors affect the distribution and attributes of assemblages and artifacts in an exposed landscape with multiple occupation events.

This model evaluates the relationships between occupational intensity, movement tortuosity, probability of recycling behaviors, selection criteria, and the proportion of recycled artifacts in surface assemblages. This model also evaluates the relationships between the proportion of recycled artifacts in assemblages with occurrence of behavioral events at particular landscape positions.

**Entities, state variables, and scales**

This model consists of four primary entities: mobile agents, flake agents, nodule agents, a landscape grid with grid squares and layers that act as storage containers and behavior recorders. The model also keeps track of the global current “age” of the model, where each time step is arbitrarily 100 time-units younger than the previous time step^[[1]](#footnote-1)^.

At the beginning of each model run a Grid is created with a certain number of rows and columns. This Grid contains a double array of Squares that are identifiable by a row and column number. Each Square contains an array list of Layers that can be used to represent different geological layers^[[2]](#footnote-2)^. Layers are represented primarily by a date integer and lists of nodules and flakes. Layers also track behaviors that happen at a particular location at a particular moment in time.

The agents are represented by an identification number, a specific technology type coded as an integer, an empty nodules list, and an empty flakes list. Additionally, agents keep track of their current location and whether they are within the gridded landscape. The group agents also act as transport vehicles for the artifact, discarding these objects at site locations in the environment.

Nodules are modeled as 20-sided polygons, with an arbitrary volume (100000 squared units) and surface area (11091.8 cubed units), following the values used by Davies et al (2018). Nodules also have a list of flakes that can be removed from the nodules by agents. Each of these flakes has a specific size (see below) and a volume. Flake volume determined by a percentage of the nodule volume (4%) and the size of the flake. Nodule surface area is divided by 20 and multiplied by the size of the flake to determine flake surface area. Nodules also have an ordered list of groups who have modified the artifact and an ordered list of the technological type of modifications made.

Flakes are represented by an integer that denotes the current stage of manufacture, an ordered list of groups who have modified the artifact, and an ordered list of the technological type of modifications made. Flakes have a specific size (up to *maxFlakeSize*), volume (see above), and shape (either blade or flake). Flakes also keep track of their initial discard year, whether they have been recycled, and whether they are still usable.

**Process overview and scheduling**

**
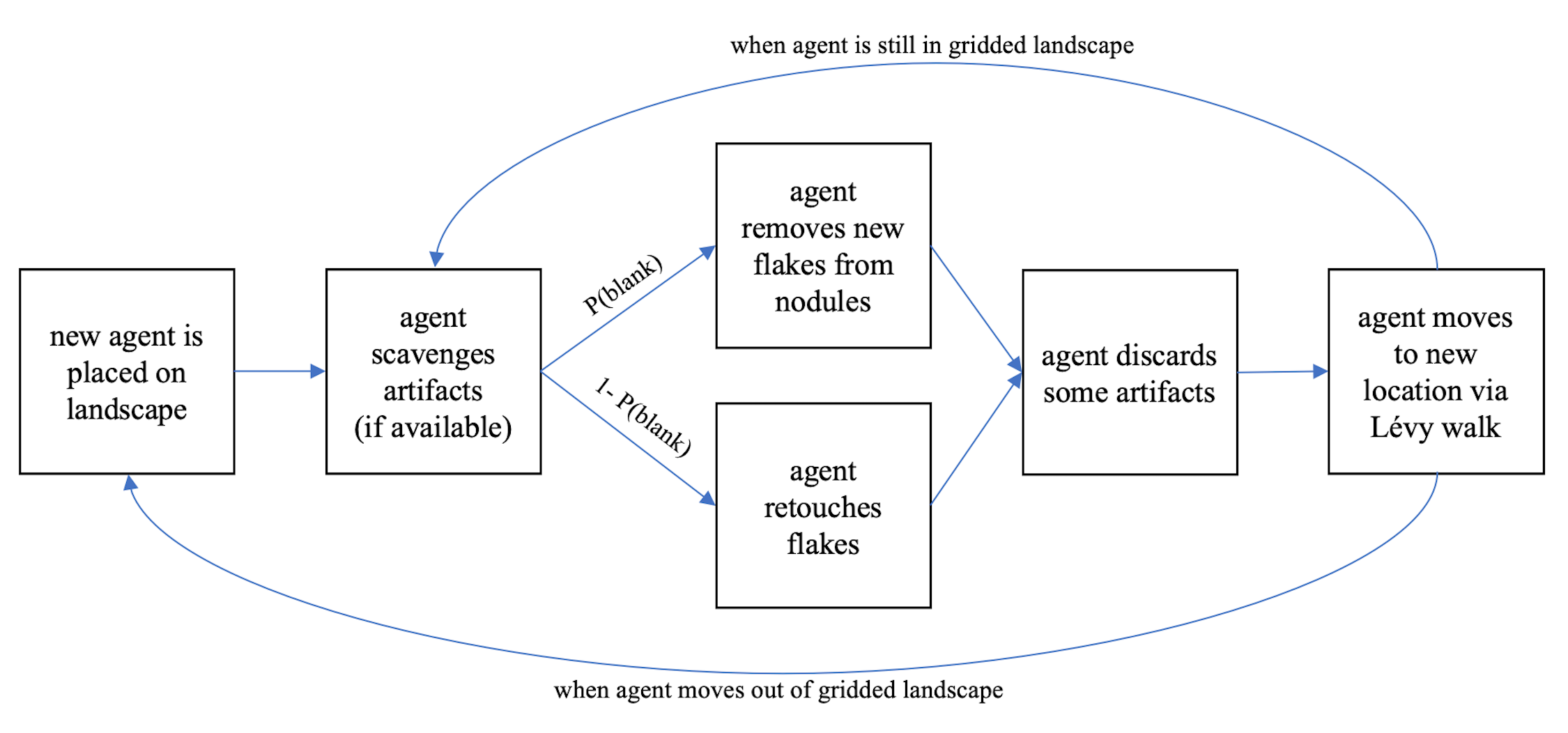
**

*Figure 1. Flow chart of process scheduling for recycling model.*

At each step of the model, the current agent first checks to make sure it is still within the gridded landscape. If it is not, the model moves onto the next agent in the list of agents, placing that new agent at a random place on the landscape.

When an agent is occupying the gridded landscape, it will first check whether there are objects available for scavenging at the Square it is currently occupying. The agent will scavenge available objects with probability *scavengeProb*. The types of objects that are collected are determined by the selection parameters (*flakePref, sizePref, strictSelect*).

After scavenging, the agent checks whether it is holding any objects. If so, it will make blanks or retouch flakes with a certain probability; the parameter *blankProb* determines how frequently agents make blanks. If the agent does not have any nodules, it will “find” new ones to refill its carried artifacts up to *maxArtifactCarry* capacity. In this way, the model simulates locally available raw materials. The *maxUseIntensity* parameter limits the number of blanks that an agent create (unless there are not enough flakes left on nodules to remove) or flake retouches an agent can perform during a given time step.

Once the agent has scavenged (if possible) and performed manufacturing actions, it will discard artifacts. The agent will first drop any exhausted artifacts (i.e., nodules with no flakes remaining). The agent will also drop additional artifacts to ensure the *maxArtifactCarry* parameter is not exceeded. The types of artifacts that are dropped are determined by the selection preference parameters (*flakePref*, *sizePref*, *strictSelect*).

Finally, the agent will move to a new landscape position via a Lévy walk.

Simulations continue until all initialized agents have entered and exited the landscape.

**Design concepts**

*Basic principles*

The recycling model is a simplified representation of stone tool scavenging, manufacture, and discard behaviors. The forager moves on a landscape and collects artifacts to recycle where possible with a certain probability and performs simplified manufacturing behaviors with a certain probability, roughly simulating what occurs at archaeological sites. The forager moves with a degree of movement tortuosity across the landscape. Greater tortuosity results in more redundant use of place, creating more opportunities for local discard and fewer opportunities for removing objects from the landscape.

*Emergence*

The assemblage composition of layers within a grid square occurs due to the interaction of individual agent behaviors with individual flake and nodule objects.

*Interaction*

Agents interact with the gridded landscape by adding flakes and nodules to assemblages and by collecting flake and nodules from preexisting assemblages. Agents also interact with artifacts by modifying them.

*Stochasticity*

Agents move randomly via Lévy walks, giving no preference to one location over another.

*Observation*

Data recording happens during each step of model run. The model itself tracks the number of scavenging events that occur at each time step. Agents keep track of each location they have occupied. Flakes and nodules keep track of the model year when they are first discarded into the landscape. Flakes and nodules also keep track of whether they have been recycled. Layers/Squares keep track of the following information:

1. Number of encounters, or how many times the square has been occupied
2. Number of scavenging events
3. Number of discard events, how many times objects have been discarded at that location
4. Number of manufacturing events, how many blank production or retouch events occur at that location
5. Number of retouch events, how many flake retouch events occur at that location

At each time step, the model records:

1. The number of scavenging events that occurred during that time step
2. The number of discard events that occurred during that time step
3. The number of recycled objects created during that time step
4. The number of flakes retouched during that time step
5. The number of blanks produced during that time step
6. The total number of recycled items currently on the landscape;
7. The total number of assemblages, or grid squares containing at least one object;
8. The total number of encounters summed for all grid squares;
9. The total number of discard events summed for all grid squares;
10. The total number of manufactures and retouches summed for all grid squares;
11. The overall cortex ratio of all objects on the landscape;
12. The recycling incidence of the landscape, which is calculated as the ratio of recycled objects to the total number of objects

This information is added as a row in a CSV file that is outputted at the end of model run.

At the end of a model run data is recorded for each grid square. This includes counts of the nodules and flakes, cortex ratio and recycling incidence values, number of discard events, number of scavenging events, number of encounter events, number of manufacture events, number of retouch events, and number of occupation events.

At end of a model run data for every artifact that is contained in the landscape is also recorded. For each object, the following data is recorded: the type of object (nodule or flake), initial discard year, size, volume, amount of cortex, number of groups that have interacted with the object, the first technology type used to interact with the object, the last technology type used to interact with the object, and whether the object is recycled. For flakes, the stage of the flake, which is equivalent to how many times the flake has been retouched, is also recorded.

**Initialization**

At the start of the model, a certain number of agents are created according the *totalAgents* parameter. Technology types of these agents are determined by the *overlap* parameter. If *overlap* is 1, then half of the agents will have technology type 1 and half will have technology type 2; these agents are added to a list of agents in a randomized order. If *overlap* is 2, then each agent has a unique technology number and are added to the list of agents in sequential order. The first agent in the list is randomly placed on a grid square prior to the beginning of model run.

In terms of the landscape, the size of the landscape is *n* x *n* patches. The model is initialized with every patch have the same arbitrary “age”, which is set by the *startYear* parameter.

Unique combinations used model experiments can be found here: <https://github.com/cocoemily/recycling-Java/blob/master/run-scripts/ExtendedModel-model-runs/parameters.csv>

**Table 1.** Parameter settings used for model runs.

| **Parameter** | **Setting** |
| --- | --- |
| world size | 10 x 10 |
| totalSteps | 3000 |
| overlap | [1, 2] |
| maxUseIntensity | [15, 30] |
| maxArtifactCarry | [10, 20] |
| maxFlakeSize | [1, 2] |
| maxNoduleSize | [20] |
| blankProb | [0.25, 0.5, 0.75] |
| scavengeProb | [0.25, 0.5, 0.75] |
| mu | [1, 2, 3] |
| sizePref | [TRUE, FALSE] |
| minAcceptableFlakeSize | [1, 2] |
| flakePref | [TRUE, FALSE] |
| strictSelect | [TRUE, FALSE] |
| totalAgents | [100, 200] |

**Submodels**

*Scavenging submodel*

When there is a preference for flakes (*flakePref* = TRUE), flakes are prioritized in scavenging; if there is not a preference for flakes, nodules are prioritized in scavenging. Similarly, if there is a size preference (*sizePref* = TRUE), then agents will prioritize collection of flakes that mean the *minAcceptableFlakeSize* requirements. Size selection does not occur on nodules since nodules are always modeled as 20-sided objects. Finally, if the agents are strict in their selection criteria (*strictSelect* = TRUE), then they will not collect any other objects except those that meet their preferences. If selection is not strict, then agents will prioritize scavenging according to their preferences, but will also collect other objects. The number of objects scavenged is determined by the *maxUseIntensity* parameter and how many objects the agent is currently holding; agents will scavenge (*maxUseIntensity* – number of currently held objects).

*Blank creation submodel*

If an agent is making blanks, it will remove flakes from random nodules it is holding until all the nodules have been reduced or the number of actions is equal to *maxUseIntensity*.

Each new flake that is removed from the nodule and initialized with the technological type of the agent that produced the flake. The nodule from which the flake is removed will also have the agent’s technology type number added to its list that records its history of technology types.

The agent will place all new flakes into its artifact carrying lists.

*Flake retouch submodel*

Agents will retouch random flakes they are holding until the number of actions is equal to *maxUseIntensity*.

Each flake retouch increases the stage of manufacture of the flake by 1 and adds the agent’s technology type to the list of the flake’s history of technology types.

*Discard submodel*

When discarding, agents will preferentially discard flakes and nodules that do not meet their selection criteria. This is done by first adding all objects that match selection criteria to a list and then parring that list down to the amount of objects an agent can take with it as determined by the *maxArtifactCarry* parameter. If the list is larger than *maxArtifactCarry*, objects from the list will be randomly chosen for discard along with any objects that did not match the selection criteria. If the list is smaller than maxArtifactCarry, only objects that did not match the selection criteria will be discarded.

Discarded objects are removed from the agent’s artifact lists and added to the Layer’s list of artifacts at the current position the agent is occupying.

*Movement submodel*

After initial random placement, agents perform Lévy walks within the gridded landscape until they step beyond the limits of the grid. This model uses a similar methodology to the one outlined for FMODEL (Davies et al. 2018). The direction that the agent faces is chosen randomly from degrees 0 through 360. In Lévy walks, the probability of a step length (l) is determined by equation 1:

$P\left( l \right)=l^{-\mu}$ (Eq. 1)

where *P(l)* is the probability of a step length, *l* (Tsallis 1997) and μ (mu) is a model parameter (see values of mu in Table 1). Reorganizing this equation allows for randomly drawing step lengths via equation 2:

$l={P(l)}^{-1/\mu}$ (Eq. 2)

where the probability of the step length *P(l)* is generated as a random number.

The heading and the step length are then used to determine a new x,y location within the gridded landscape (see Figure 2).


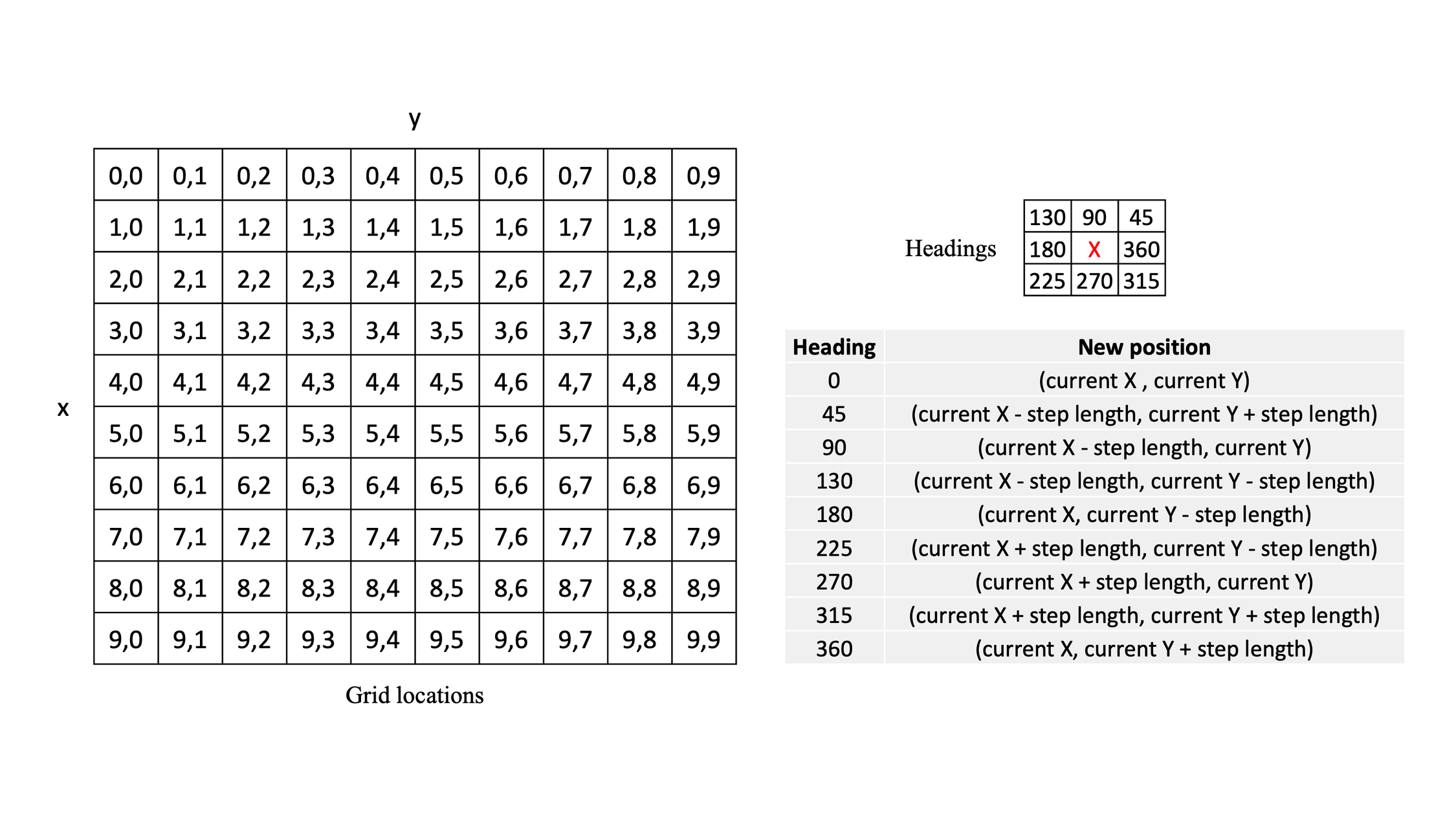


*Figure 2. Diagram of the gridded landscape, specifying all the x,y location pairs (left). Equations (grey box) for determining an agent’s new position on the landscape based on a heading from current location (marked with the red X) and the step length.*

**References**

Davies, B., Holdaway, S., & Fanning, P. C. (2018). Modeling Relationships Between Space, Movement, and Lithic Geometric Attributes. *American Antiquity*, *83*(03), 444–461. https://doi.org/10.1017/aaq.2018.23

Grimm, V., Berger, U., Bastiansen, F., Eliassen, S., Ginot, V., Giske, J., et al. (2006). A standard protocol for describing individual-based and agent-based models. *Ecological Modelling*, *198*(1), 115–126. https://doi.org/10.1016/j.ecolmodel.2006.04.023

Grimm, V., Berger, U., DeAngelis, D. L., Polhill, J. G., Giske, J., & Railsback, S. F. (2010). The ODD protocol: A review and first update. *Ecological Modelling*, *221*(23), 2760–2768. https://doi.org/10.1016/j.ecolmodel.2010.08.019

Grimm, V., Railsback, S. F., Vincenot, C. E., Berger, U., Gallagher, C., Deangelis, D. L., et al. (2020). The ODD protocol for describing agent-based and other simulation models: A second update to improve clarity, replication, and structural realism. *Journal of Artificial Societies and Social Simulation*, *23*(2). http://eprints.bournemouth.ac.uk/33918/. Accessed 1 August 2023

Tsallis, C. (1997). Lévy distributions. *Physics World*, *10*(7), 42. https://doi.org/10.1088/2058-7058/10/7/32

1. This numbering of time steps is only used to help distinguish between “ages” of artifacts and is not intended to relate to any particular unit of time. [↑](#footnote-ref-1)
2. This function is not used in the model described in this paper. [↑](#footnote-ref-2)
