# Supplementary figures and images for: Exploring the impact of mobility and selection on stone tool recycling behaviors through agent-based simulation
Source: PLoS One. 2023 Nov 9;18(11):e0294242. doi: 10.1371/journal.pone.0294242 (PMC10635449; doi:10.1371/journal.pone.0294242)

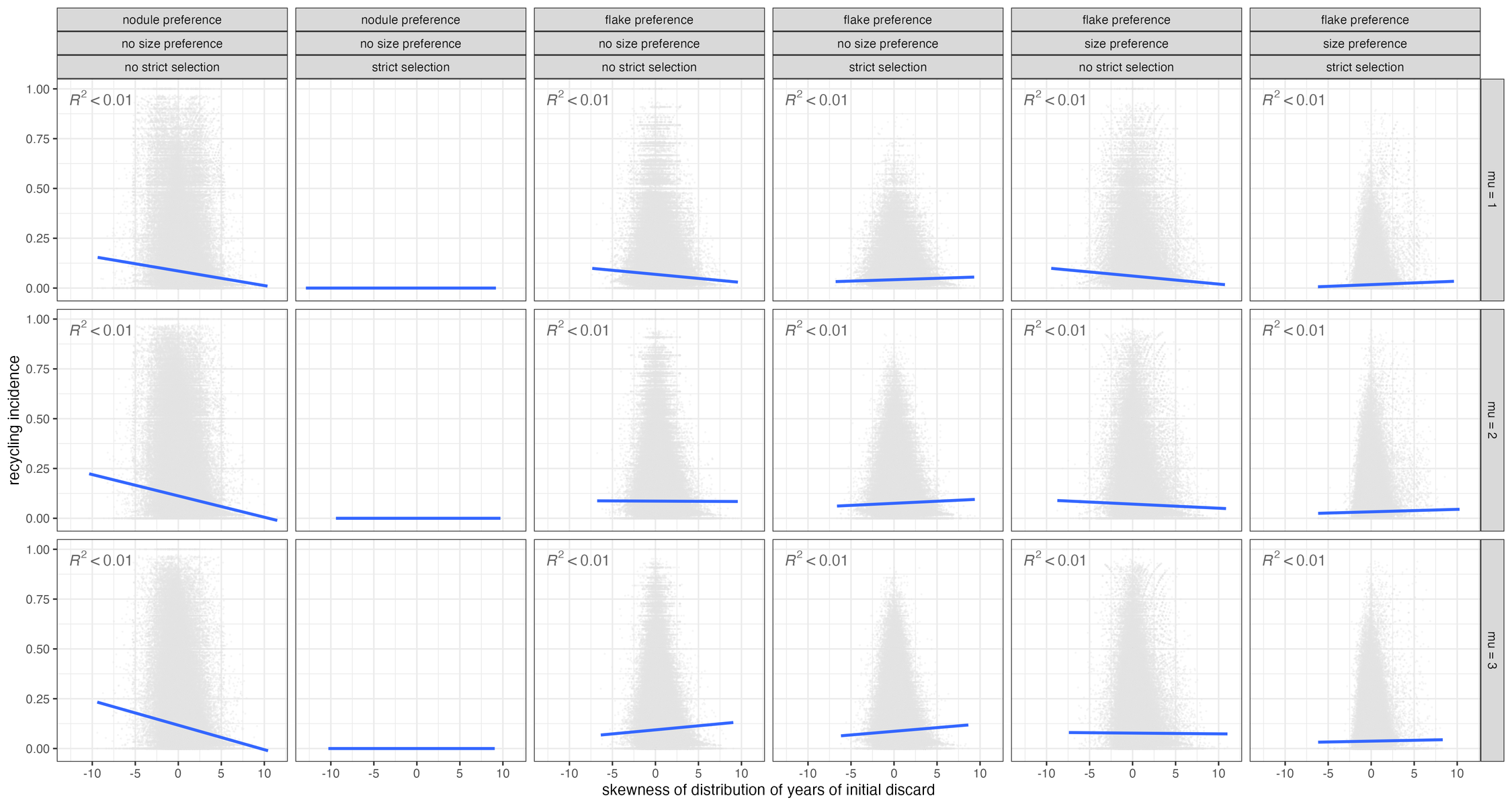

Supplement: S1 Fig — Results show for model runs when agents have one of two technology types (overlap = 1) and only 100 agents occupy the landscape during model run. Negative skew indicates artifacts in the assemblage are first discarded earlier in model run. Positive skew indicates artifacts are discarded later in model run. Linear relationship shown by dark blue line. R squared values given in upper left corner of each panel. (TIF) [file pone.0294242.s002.tif]

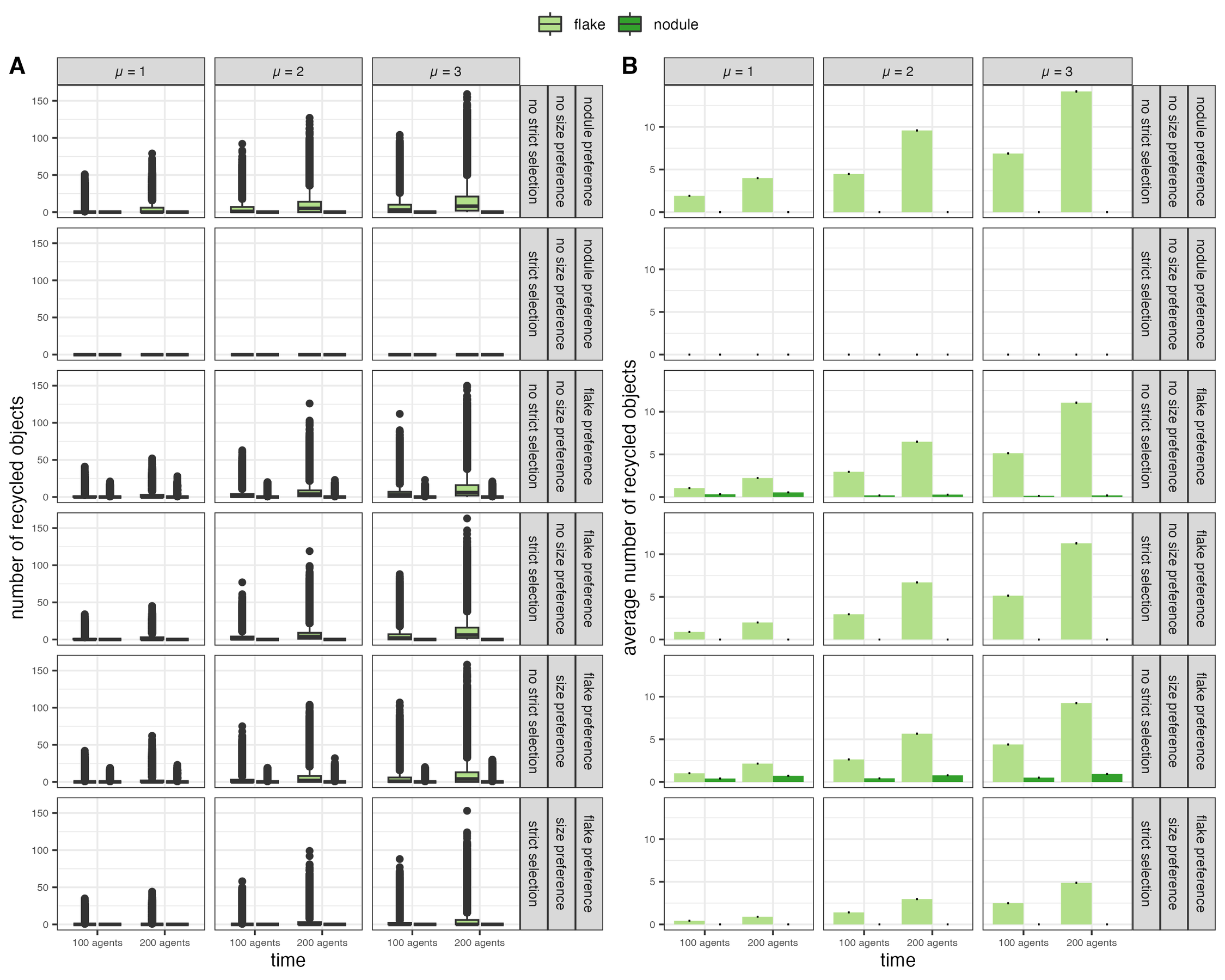

Supplement: S2 Fig — Results shown for model runs when agents have one of two technology types (overlap is 1). Error bars in panel B represent the 95% confidence intervals on the average per model run. (TIF) [file pone.0294242.s003.tif]

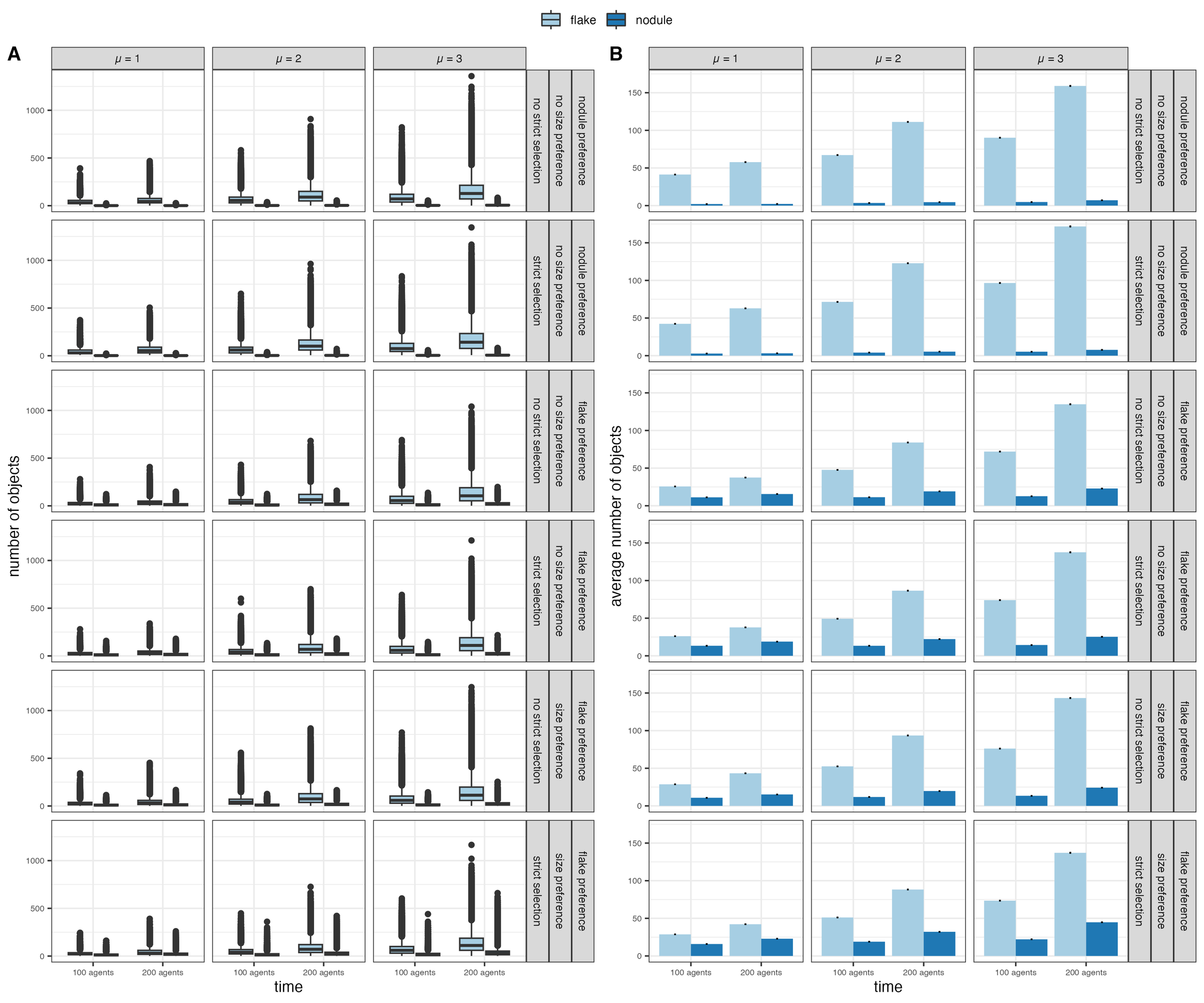

Supplement: S3 Fig — Results show for model runs when agents have one of two technology types (overlap is 1). Error bars in panel B represent the 95% confidence intervals on the average per model run. (TIF) [file pone.0294242.s004.tif]

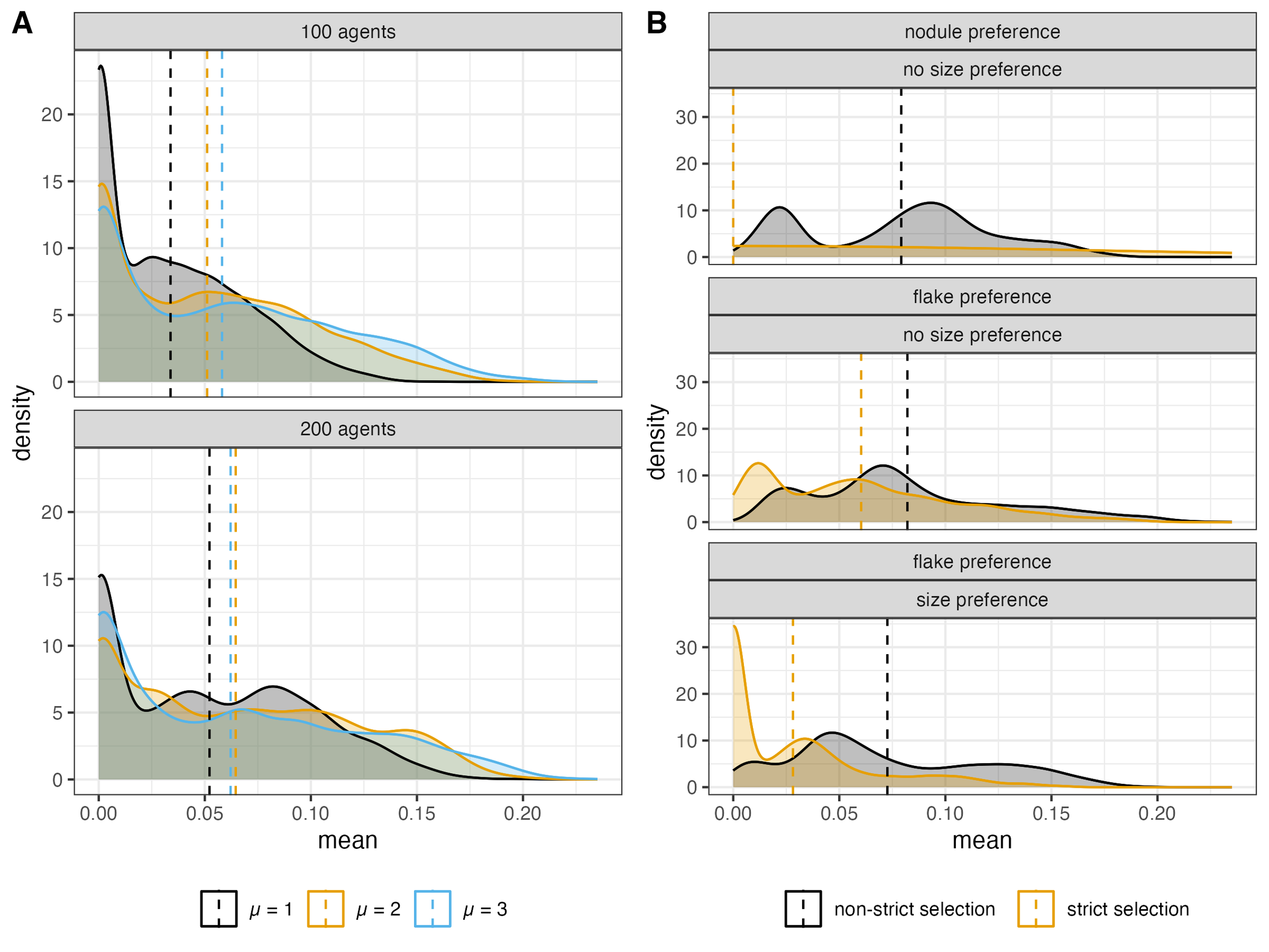

Supplement: S4 Fig — Dotted lines show the mean value of the distributions. (TIF) [file pone.0294242.s005.tif]

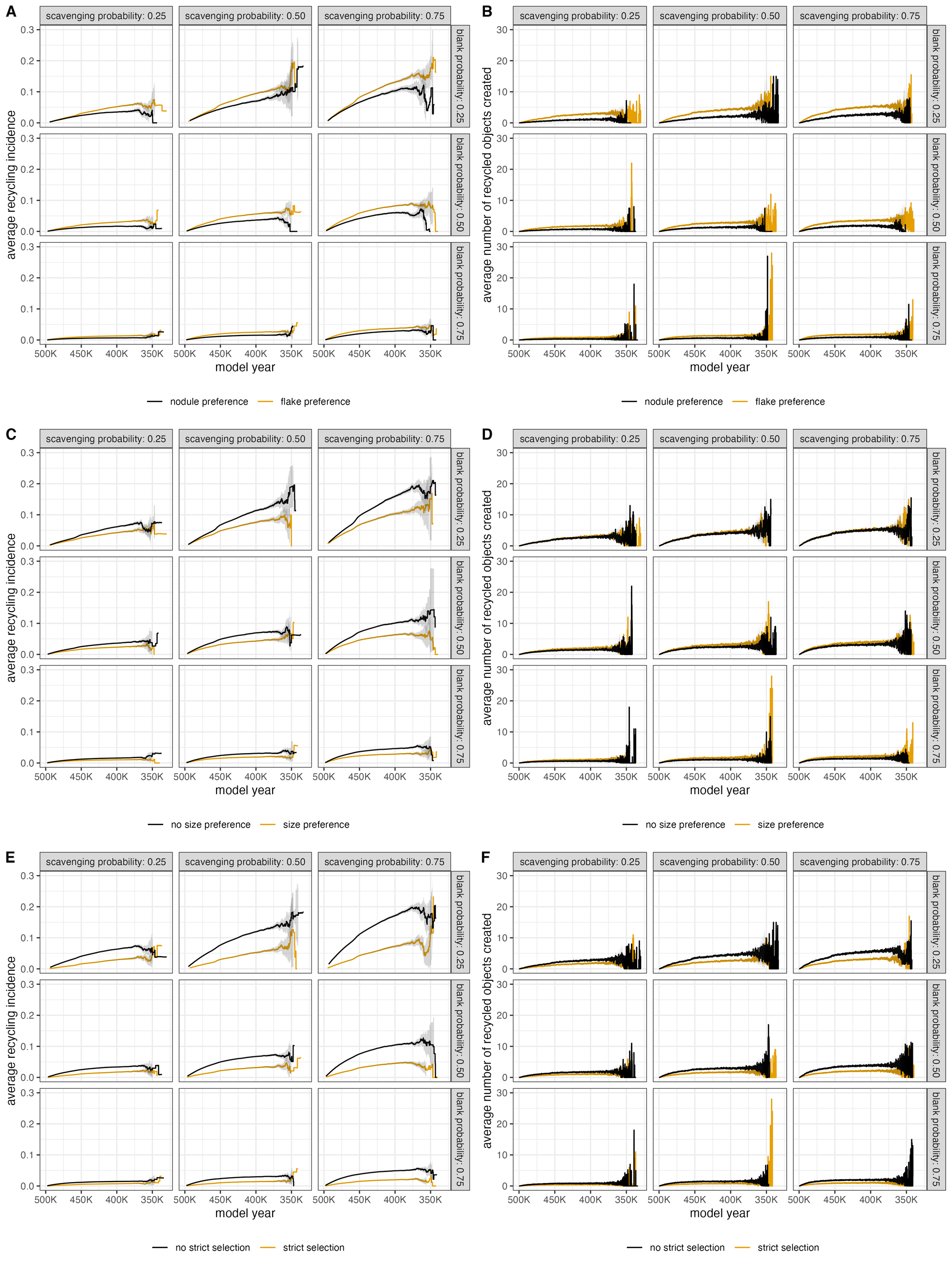

Supplement: S5 Fig — Trend lines for model runs with an overlap parameter of 1 and 100 agents. (TIF) [file pone.0294242.s006.tif]

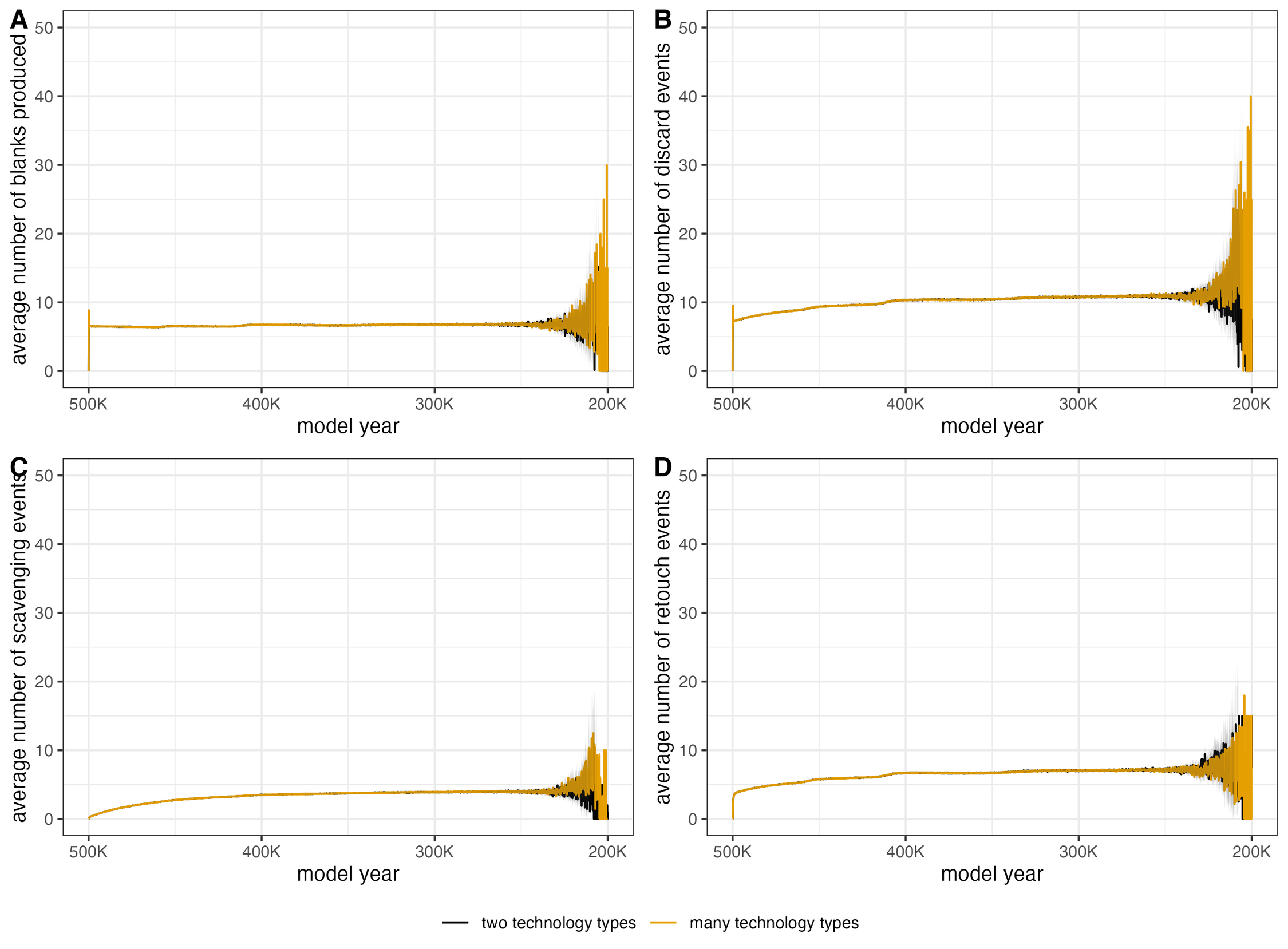

Supplement: S6 Fig — Behavioral events include: number of blanks produced (A), number of discard events (B), number of scavenging events, and number of artifact retouches (D). (TIF) [file pone.0294242.s007.tif]

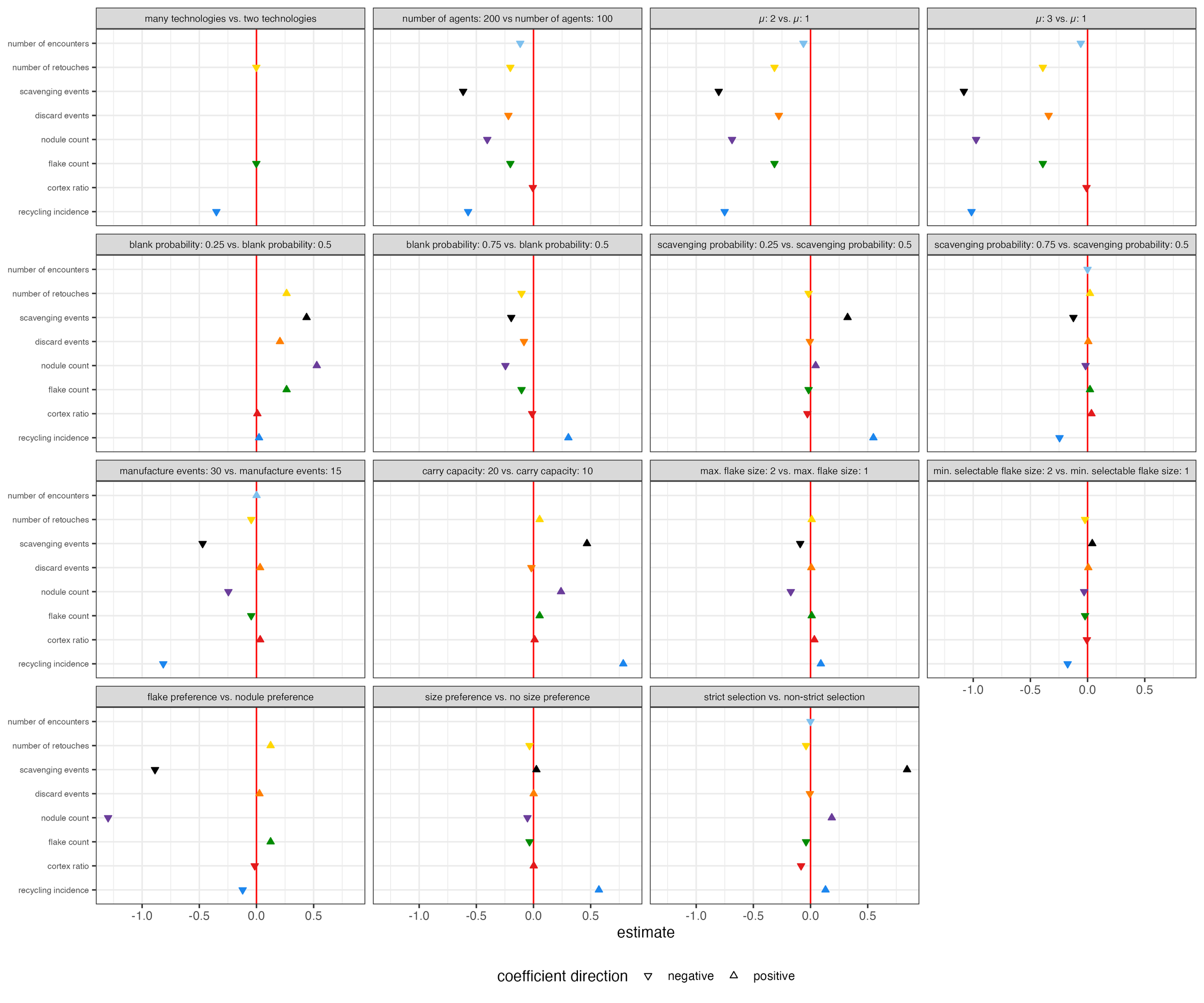

Supplement: S7 Fig — Each facet shows the effect of a different dependent variable on the COVs of each output. (TIF) [file pone.0294242.s008.tif]

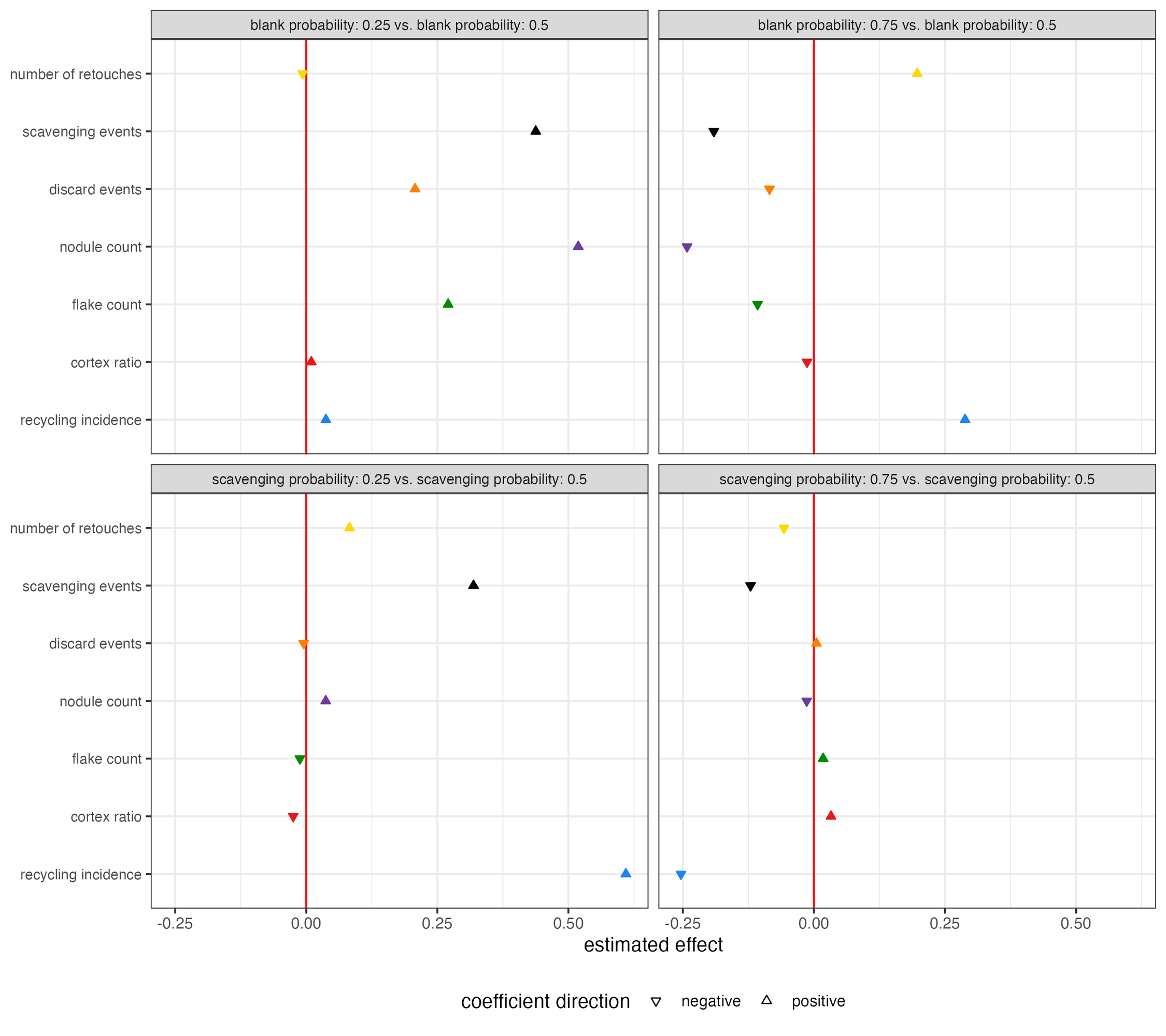

Supplement: S8 Fig — Estimated effects calculated from regression coefficients for blank probability, scavenging probability, and interaction terms between the two. (TIF) [file pone.0294242.s009.tif]

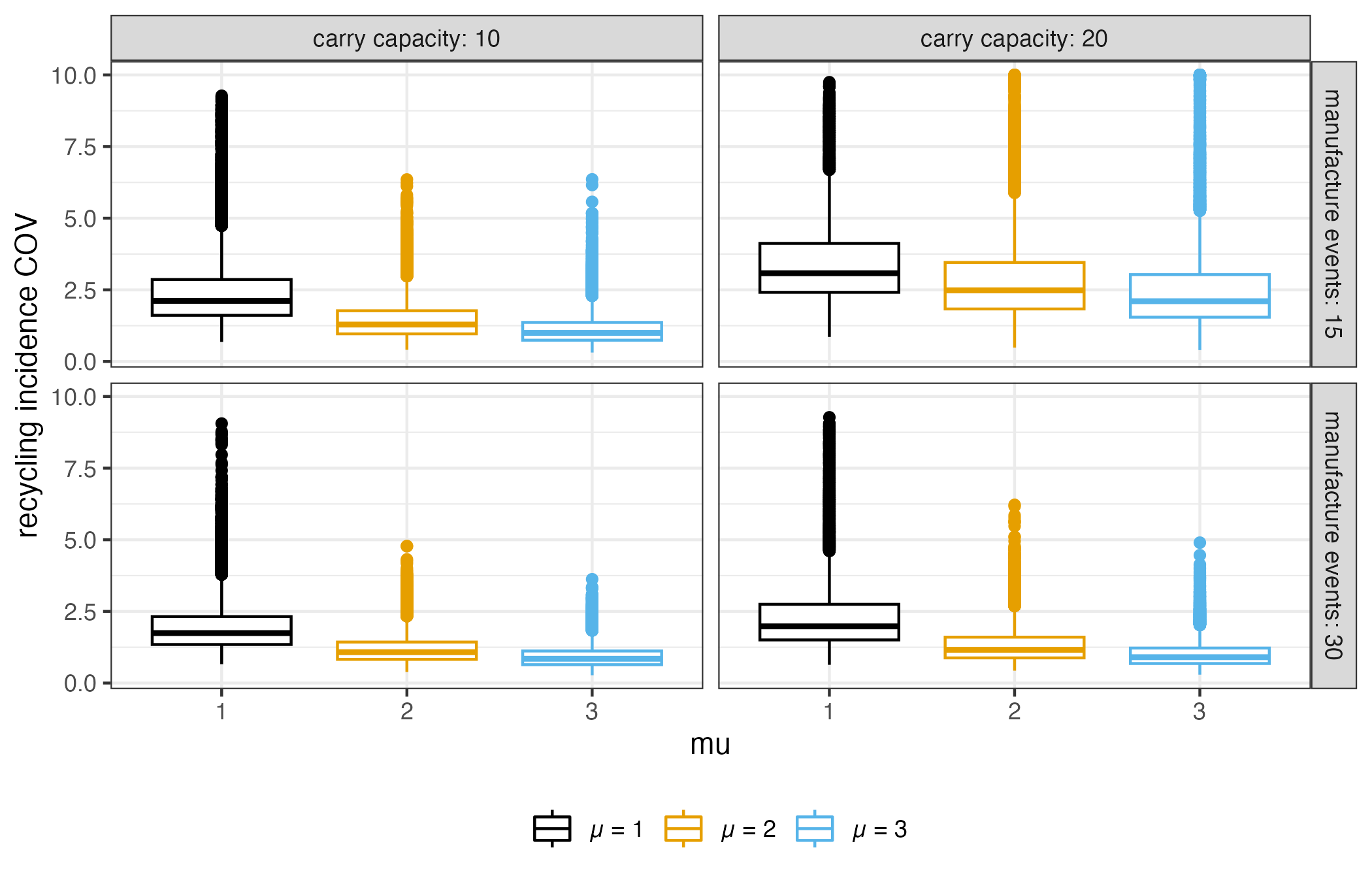

Supplement: S9 Fig — Only data from model runs where agents had one of two technology types (overlap = 1) and 200 agents occupied the landscape during a model run. (TIF) [file pone.0294242.s010.tif]

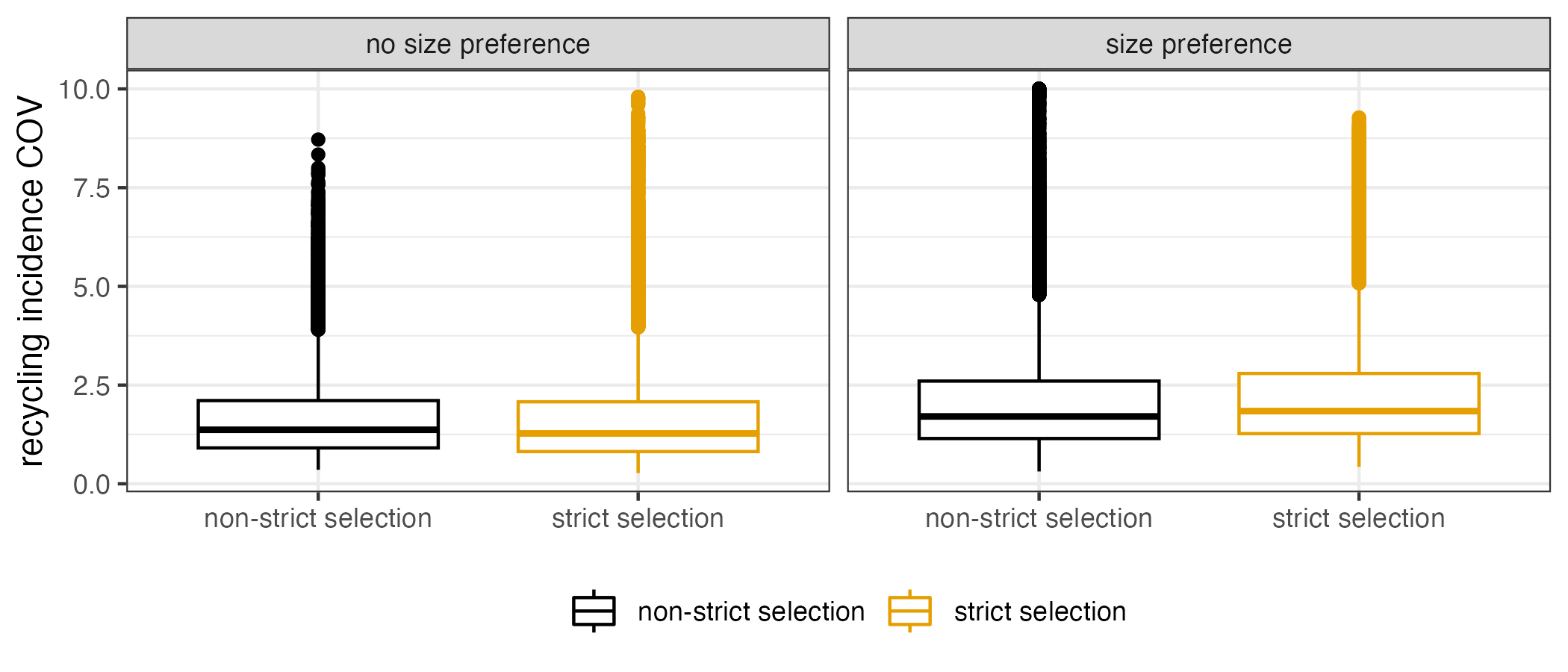

Supplement: S10 Fig — Only data from model runs where agents had one of two technology types (overlap = 1), 200 agents occupied the landscape during a model run, and flakes are preferred. (TIF) [file pone.0294242.s011.tif]

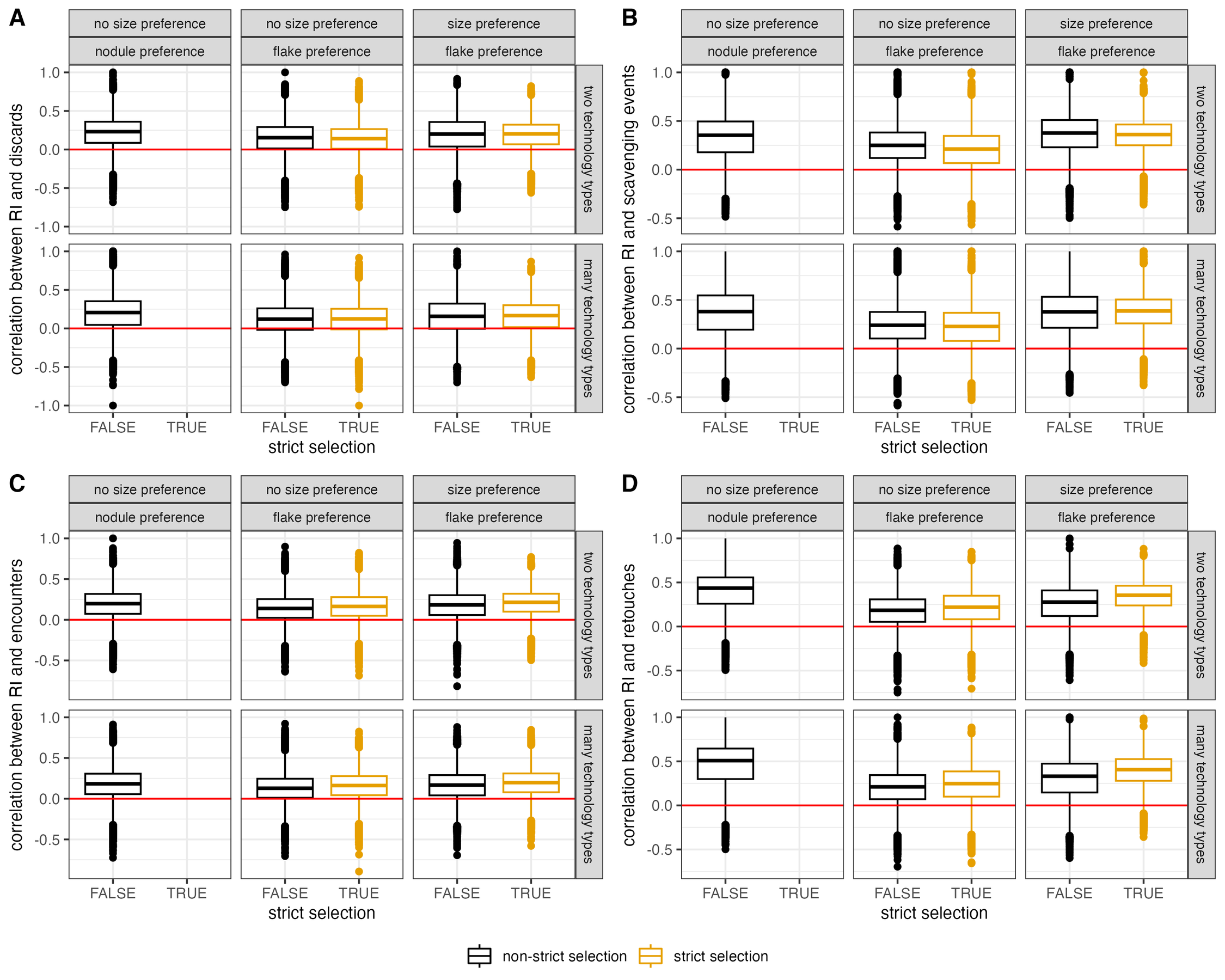

Supplement: S11 Fig — The correlations are between recycling intensity and: number of discard events (A), number of scavenging events (B), number of grid square encounters (C) and number of retouch events (D). (TIF) [file pone.0294242.s012.tif]

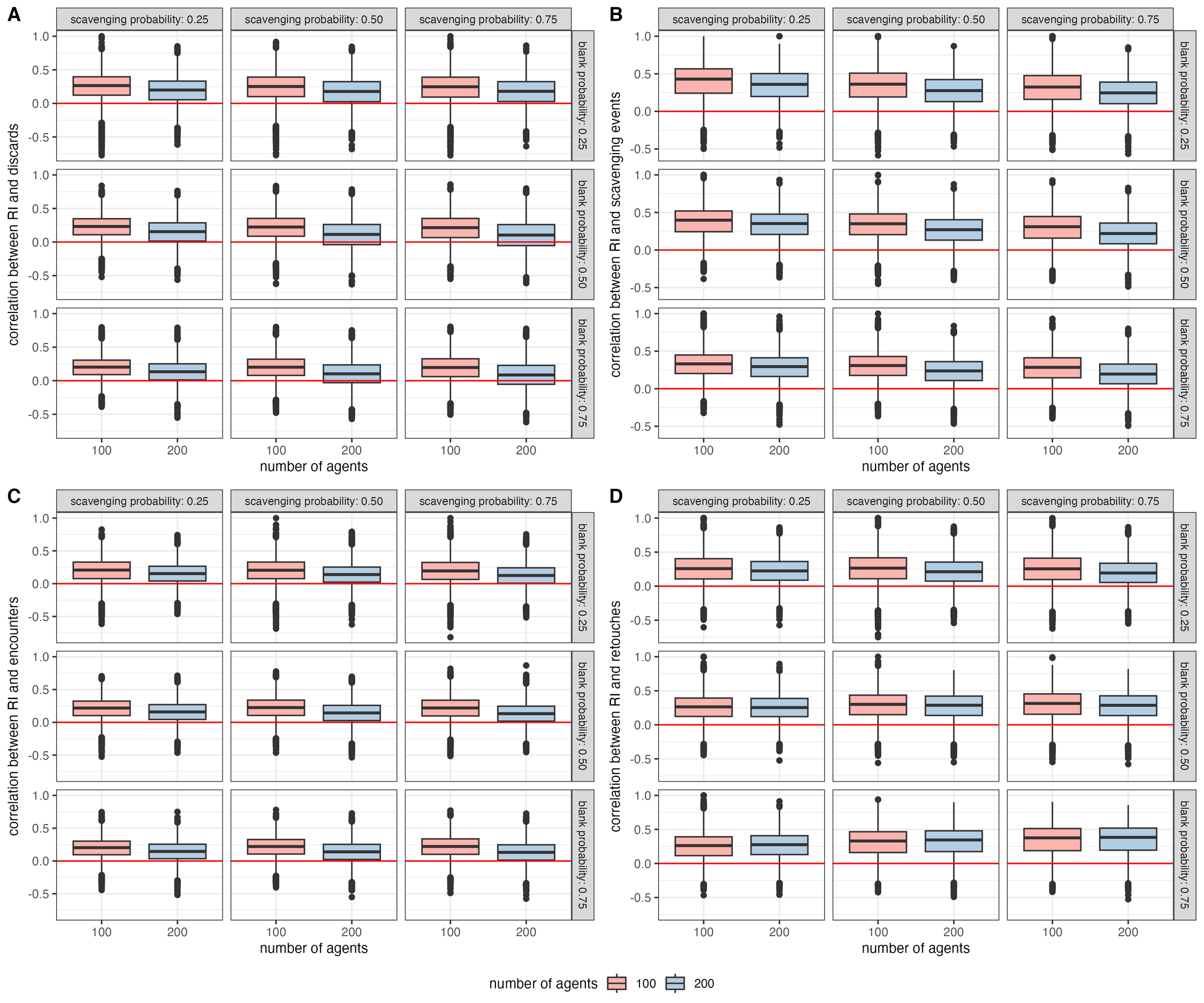

Supplement: S12 Fig — Results shown for model runs when the overlap parameter is 1. The correlations are between recycling intensity and: number of discard events (A), number of scavenging events (B), number of grid square encounters (C) and number of retouch events (D). (TIF) [file pone.0294242.s013.tif]

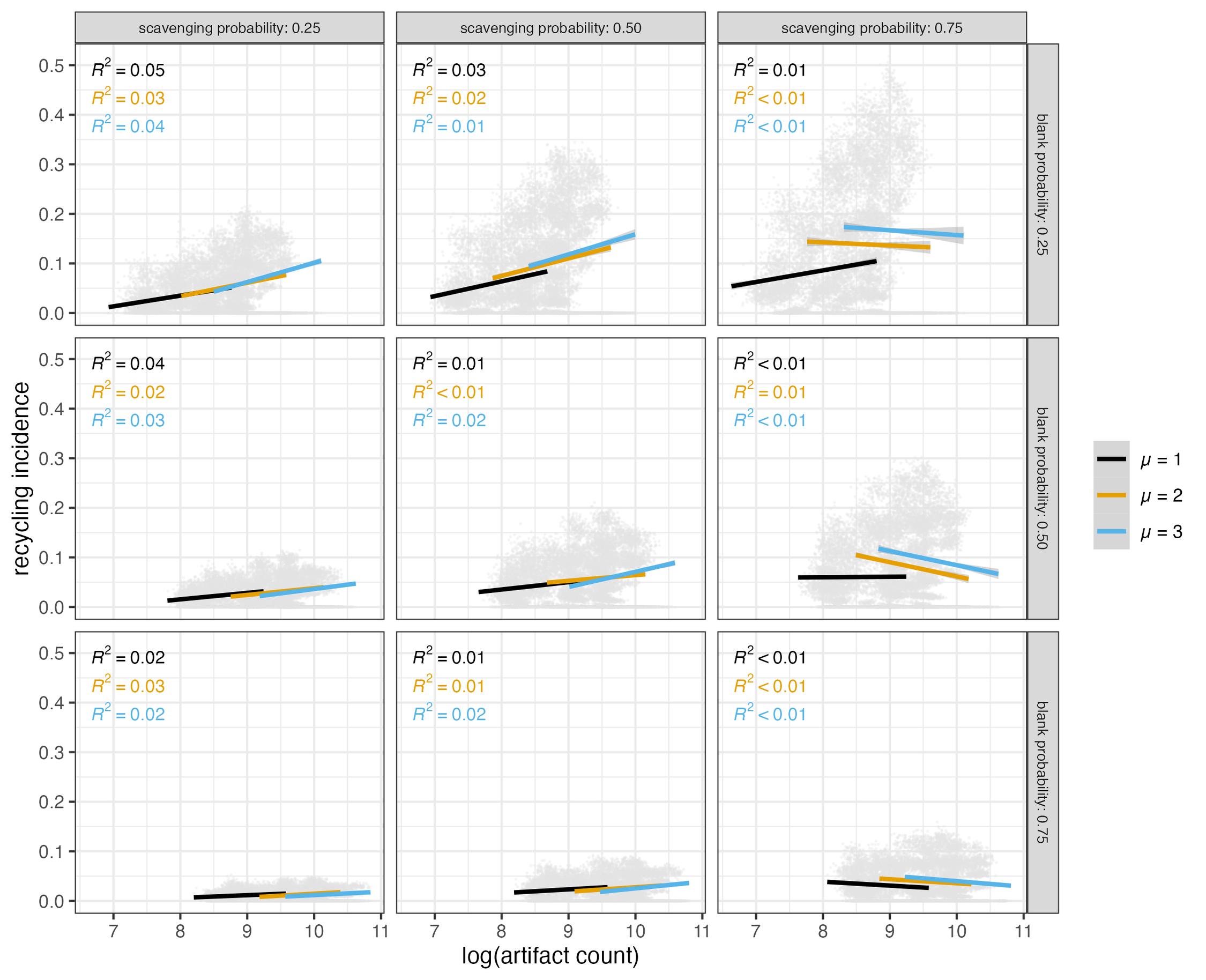

Supplement: S13 Fig — Results for model runs where agents have one of two technology types (overlap is 1) and only 200 agents occupy the landscape during model run. Linear relationship shown for each mu value. Equation and R squared value for each line given in upper left corner of each panel. (TIF) [file pone.0294242.s014.tif]

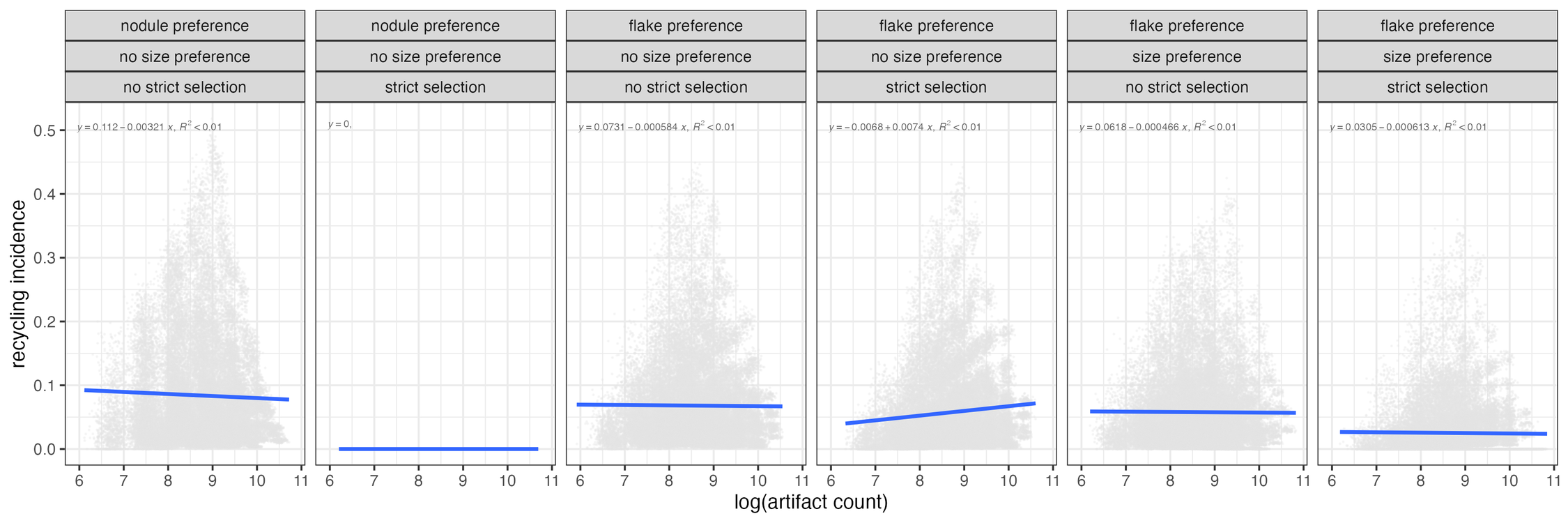

Supplement: S14 Fig — Results for model runs where agents have one of two technology types (overlap is 1). Linear relationship shown by dark blue line. Equation and R squared value for each line given in upper left corner of each panel. (TIF) [file pone.0294242.s015.tif]

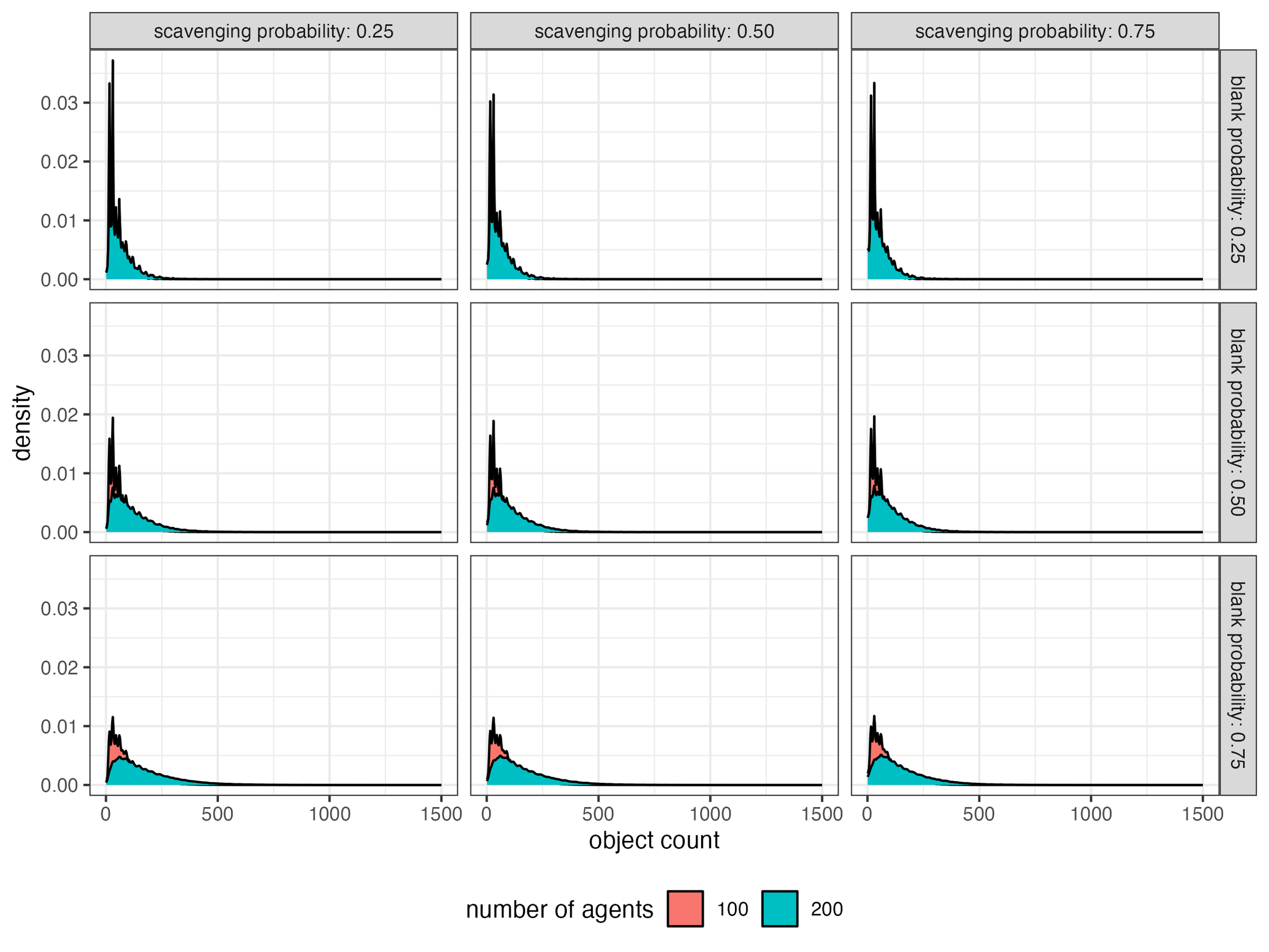

Supplement: S15 Fig — Results shown for model runs where agents have one of two technology types (overlap is 1). (TIF) [file pone.0294242.s016.tif]

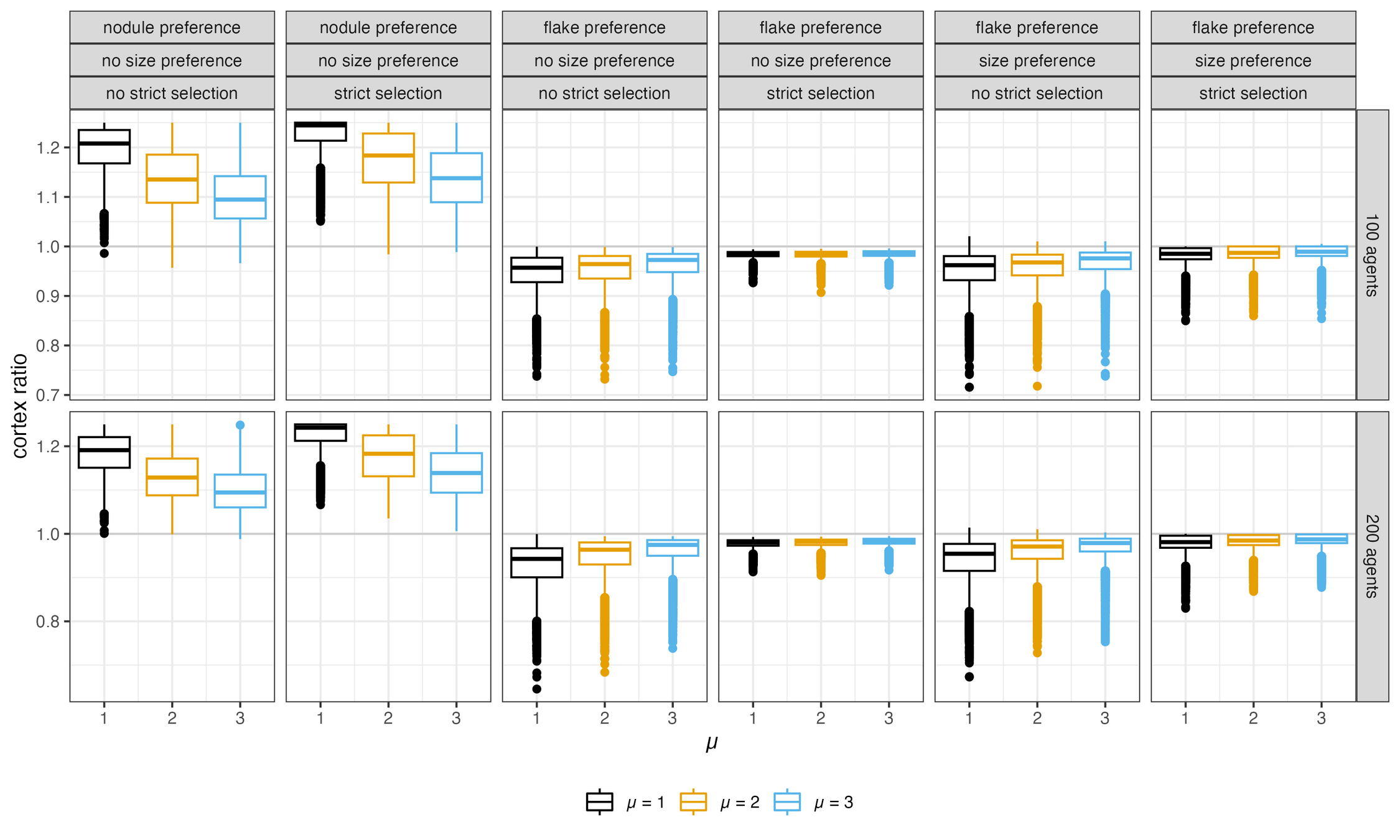

Supplement: S16 Fig — (TIF) [file pone.0294242.s017.tif]

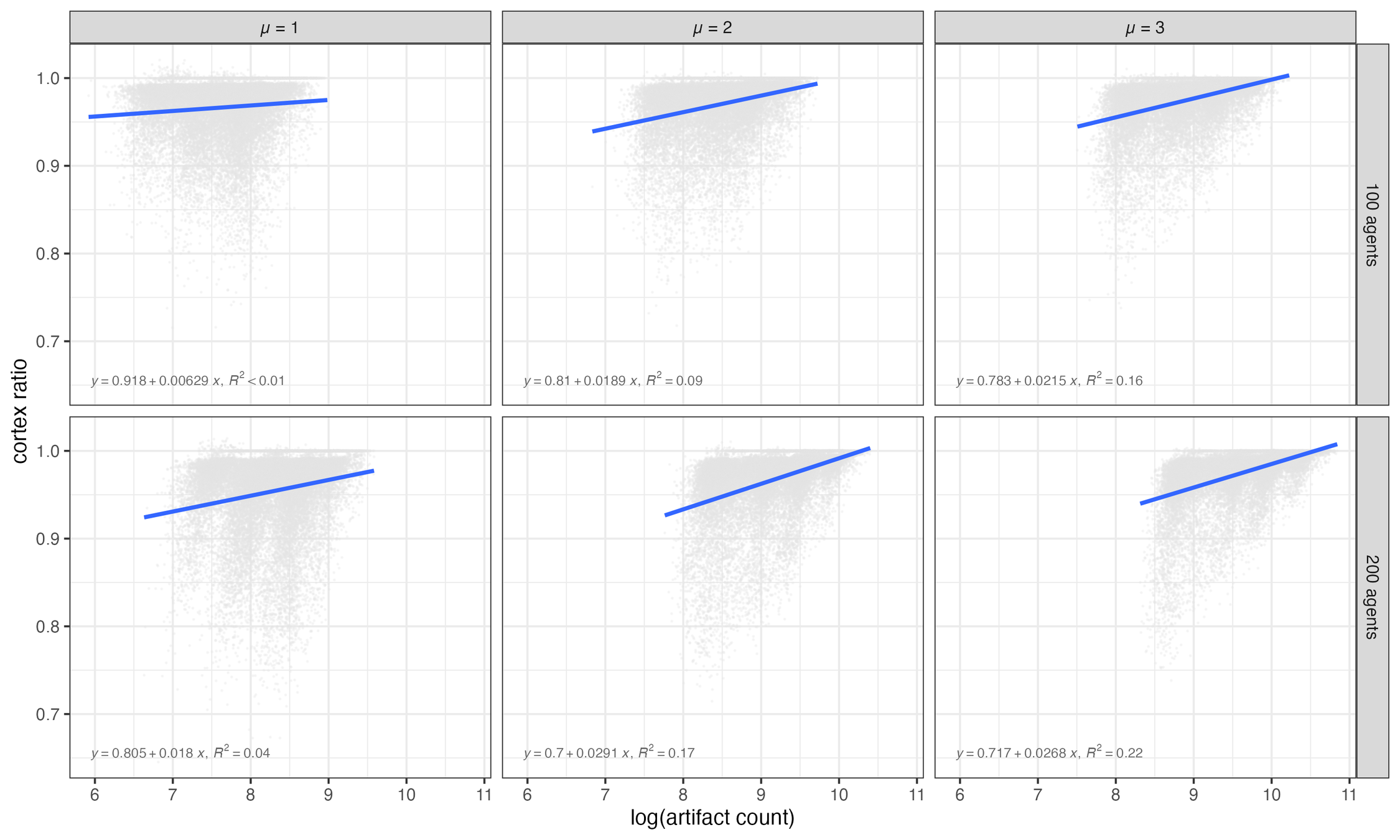

Supplement: S17 Fig — Results for model runs where agents have one of two technology types (overlap is 1) and prefer flakes (flake_preference is TRUE). (TIF) [file pone.0294242.s018.tif]

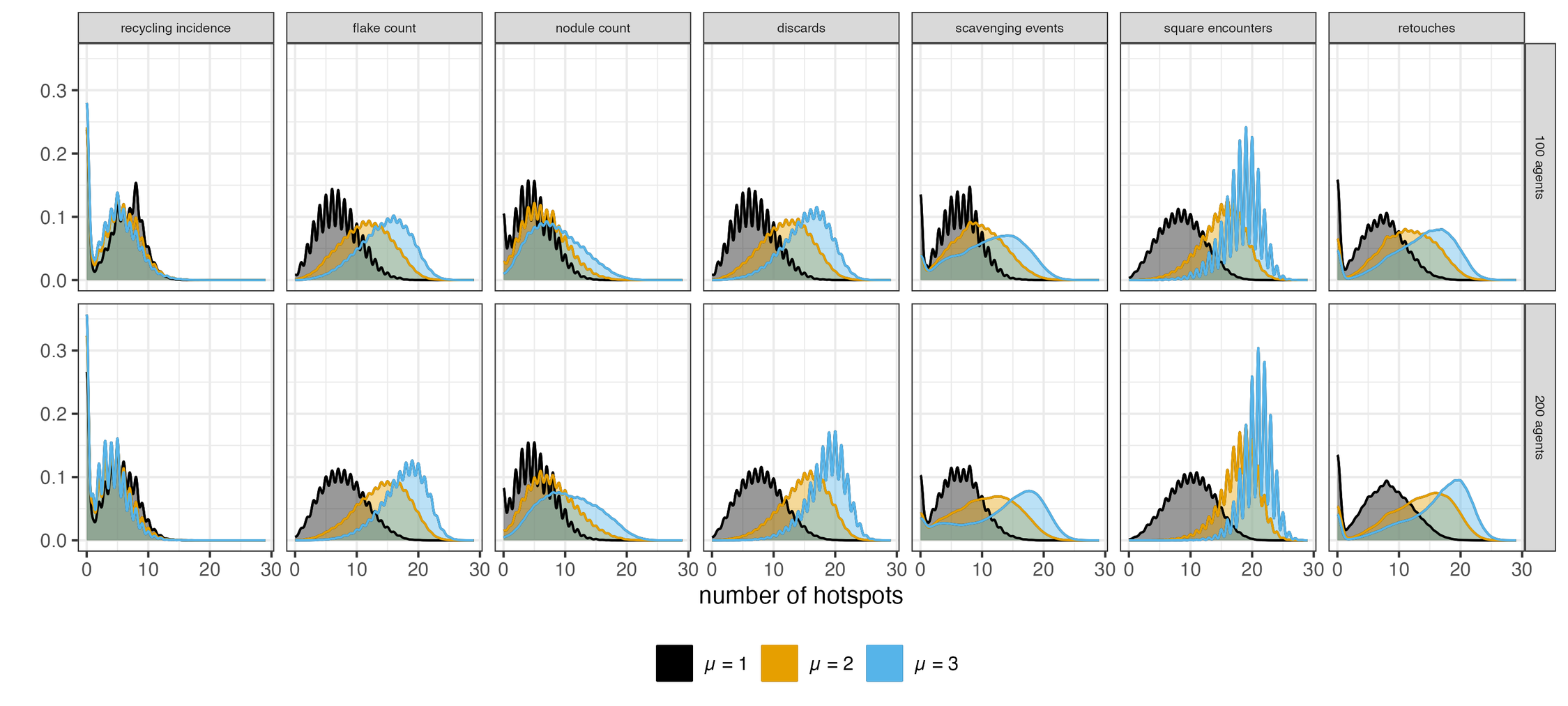

Supplement: S18 Fig — (TIF) [file pone.0294242.s019.tif]

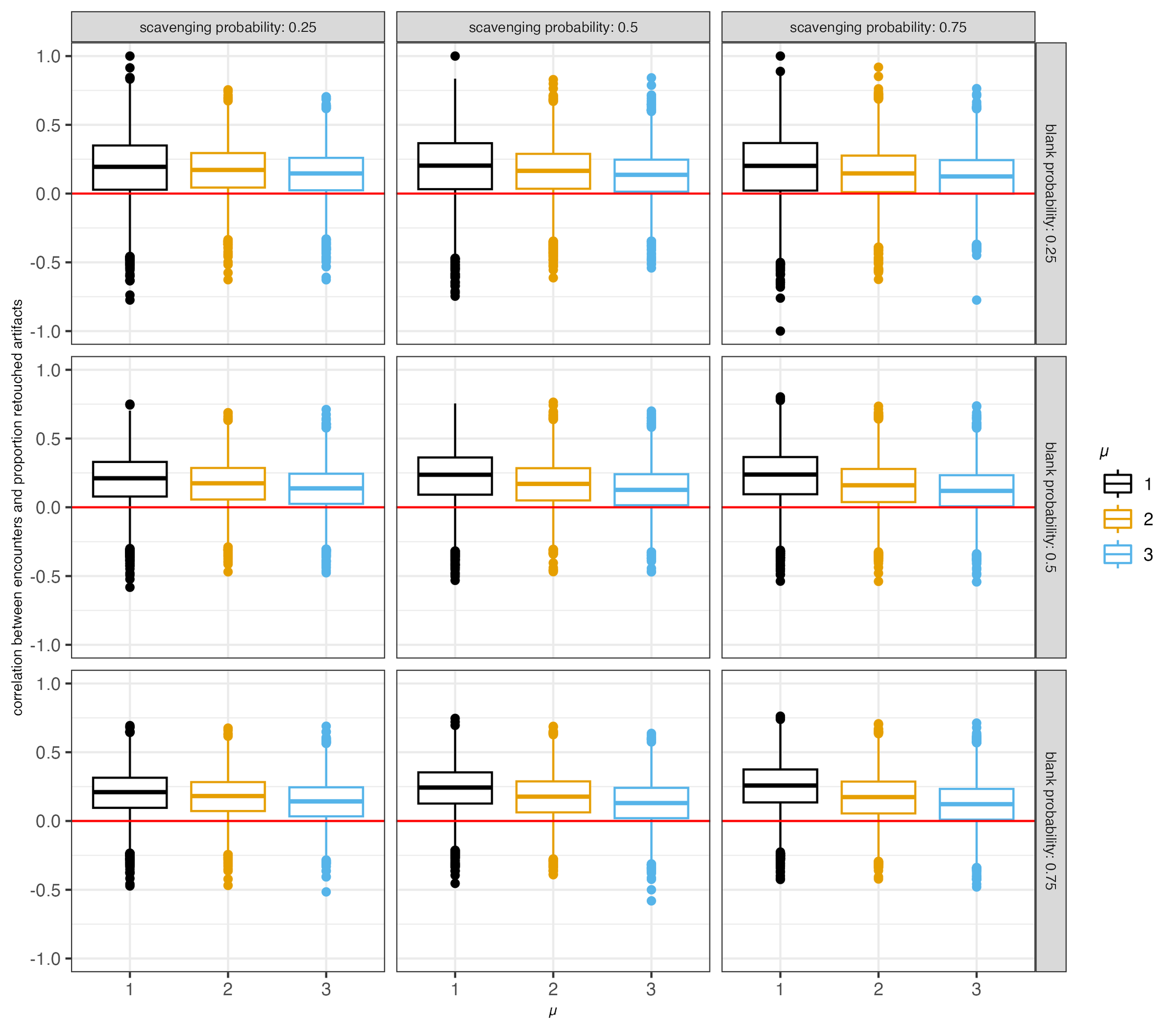

Supplement: S19 Fig — (TIF) [file pone.0294242.s020.tif]

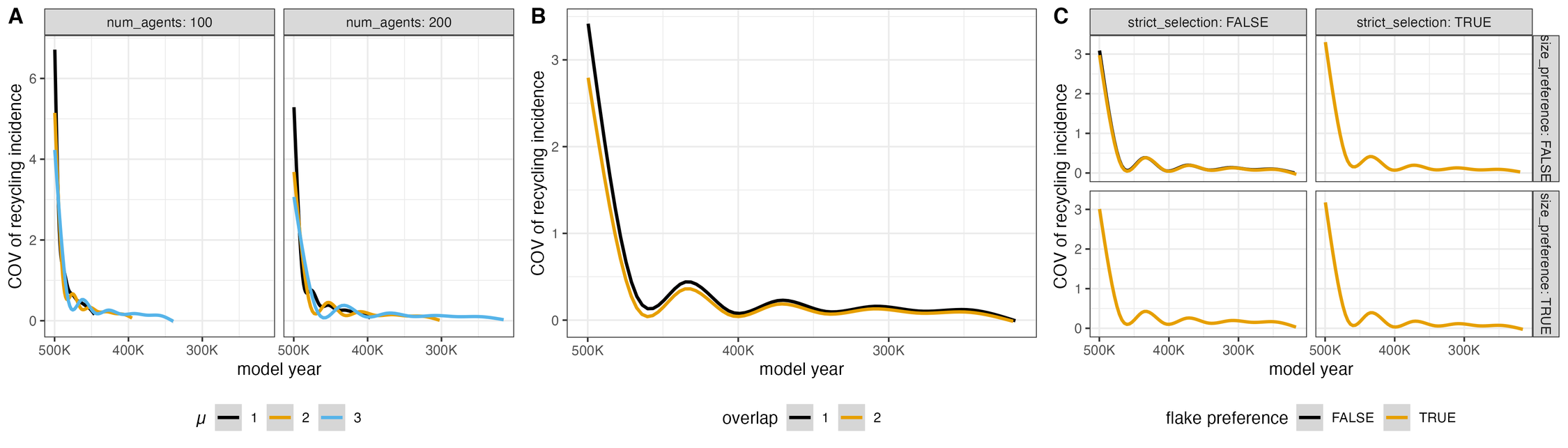

Supplement: S20 Fig — (TIF) [file pone.0294242.s021.tif]

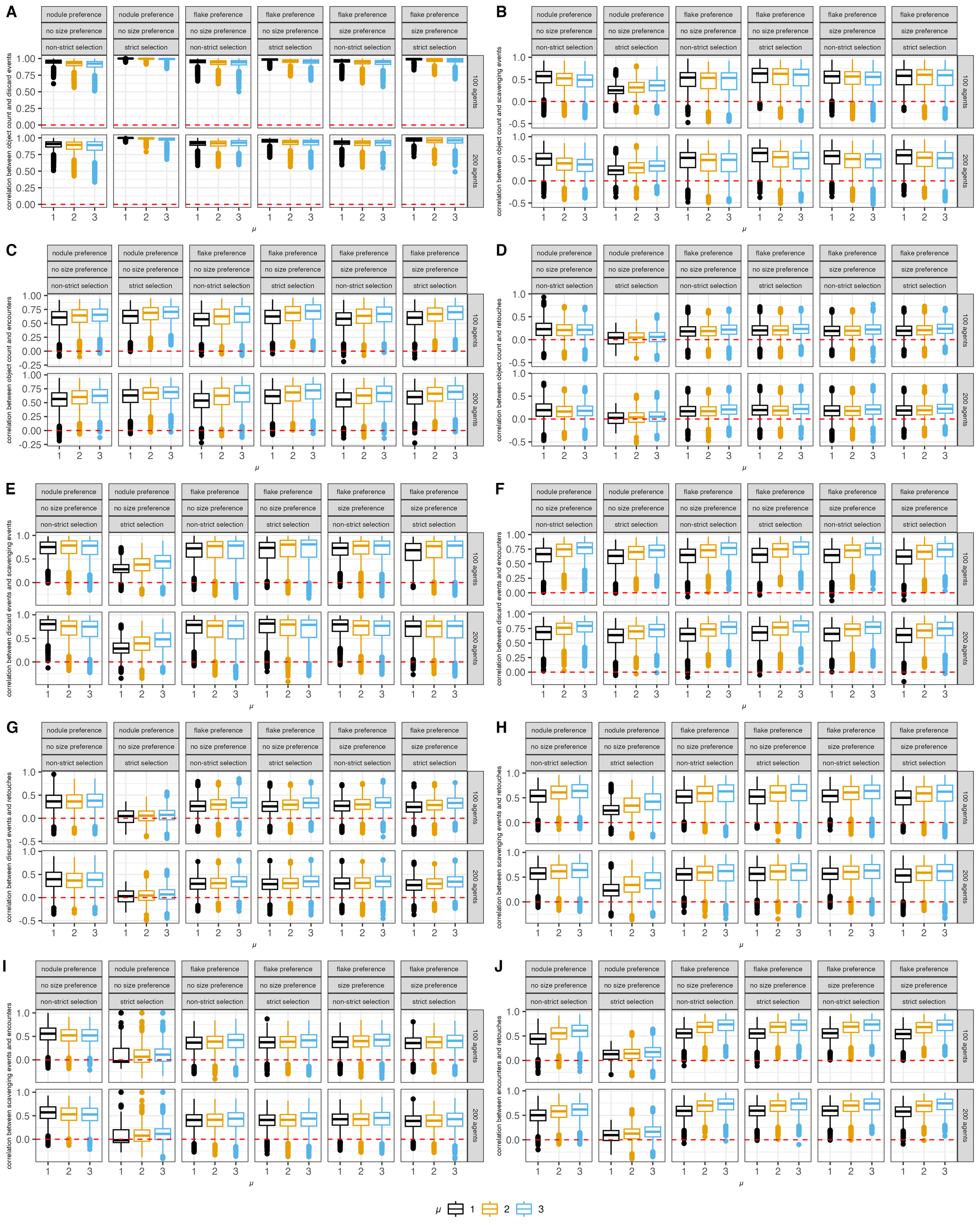

Supplement: S21 Fig — Correlations are calculated for each grid square. (TIF) [file pone.0294242.s022.tif]

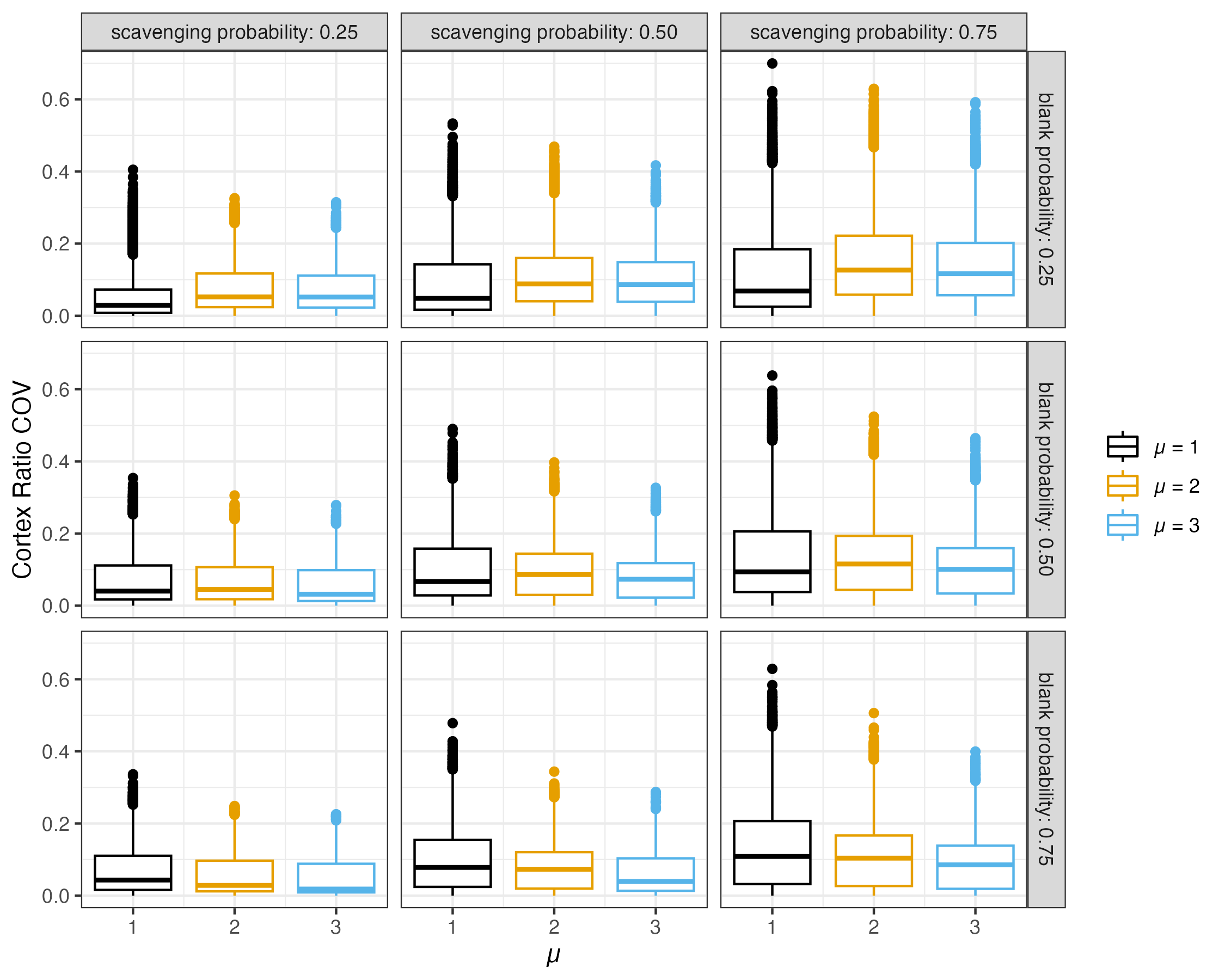

Supplement: S22 Fig — (TIF) [file pone.0294242.s023.tif]
