# Supplementary material for: Exploring the impact of mobility and selection on stone tool recycling behaviors through agent-based simulation
Source: PLoS One. 2023 Nov 9;18(11):e0294242. doi: 10.1371/journal.pone.0294242 (PMC10635449; doi:10.1371/journal.pone.0294242)
Supplement: Attachment — Submitted filename: PLoS-One_reviewer-comments.docx [file pone.0294242.s024.docx]

==============================
Both reviews are rather positive. Please do your best to adress the remarks and recommendations
==============================

I want to thank both reviewers for their constructive feedback. I also want to note that this paper is part of my dissertation, so the revisions done also include feedback from my dissertation committee.

Reviewer #1: For this review, as is my general policy for reviewing junior scholars’ work, I waive my anonymity – hello, this is Ellery Frahm, and I’ve done ABM of inter-group contacts during lithic procurement activities.

This is a well-written manuscript that considers an important question and applies ABM appropriately as a means to identify significant variables with respect to the issue of lithic recycling.

Other reviewers might suggest some other variables or conditions that they would want to see in the model, but I consider the model useful as it is. Were there other variables that I would like to have seen? Sure, and the major one would be some sort of sedimentation rate proxy that takes slowly older lithics out-of-play. However, having done ABM, I know that any sort of model is a simplified cartoon of reality, and a modeler must decide, especially during the process of testing variables and identifying key ones, what those simplifications are.

I agree that sedimentation rates would be an interested factor to add into the model. In fact, since this model is based on one that did model deposition and erosion of sediments, there are aspects of the modeling choices that would allow for that functionality to be added relatively easily. My previous paper Coco et al 2020 in JPA explores a bit how sedimentation affects the appearance of recycling, although it does not include some of the other more complex aspects of this new model.

Other reviewers might offer a critique that the ABM results are commonsensical or even obvious, like the outcome that basically states there are more recycled lithics when there is more recycling behavior. I am also fine with this aspect of the manuscript. It is important to test ideas that even seem “obvious” on the surface. And sometimes it is not only the qualitative result but also the magnitude of the effect that is an important finding.

My only suggestion for revision concerns the discussion of mu (which, to be pedantic, would ideally be the Greek letter in the text as well). There are occasional points that contrast when mu is high versus when mu is low (Lines 974-976 as one example), which leads the reader to flip back in the manuscript to re-acquaint themselves with what mu was and think again about what it means for the past – and if they find Table 2 first, then they spend time trying to think again about how low vs high movement redundancy translates to hunter-gatherer behavior. I would encourage the author to think about what h-g behaviors, as discussed (or not) in the literature, correspond to movement redundancy on the landscape and to (1) both explicitly discuss that and (2) discuss the outcomes of varying mu values in those terms. That way, the discussion would read less like “When mu is high…” and more like “When Phenomenon X is more common…,” emphasizing interest in behaviors rather than equations.

I have changed all instances of mu to μ in the text and the figures.

I agree with the reviewer that understanding the behavioral analogs for the different values of μ and therefore degree of movement redundancy is tricky. I have added in visualization of how the different values of μ affect agent movement to the description of this parameter in the “simulating agent movement” section. I have also added visualization of μ to relevant figures, so that the reader knows what changes in μ refer to.

For the results section, I try to avoid making any translation between the value of μ and hunter-gatherer behaviors. In that section, I have removed most of the wording related to movement redundancy and instead discuss μ in terms of how it changes the amount of time that each agent spends on the landscape. In the discussion section, I have reorganized things so that the first discussion of μ is accompanied by an explanation of how to conceptualize this parameter in the context of hunter-gatherer mobility.

In short, there is a lot to like about this manuscript, and I’m still digesting what some of it means and what all of the implications are, but more importantly, it is important to have a way to think about this issues in a novel way.

Reviewer #2: PONE-D-23-27739, “Exploring the impact of mobility and selection on stone tool recycling behaviors through agent-based simulation” by Coco.
Please note:
1. I consulted none of the supplementary materials.
2. Anonymity waived. This is M. Shott.

This paper follows from, but significantly extends the Coco et al. (2021) paper on which it builds. It does this by a more sophisticated modeling of mobility and by including selection criteria for scavenging. In this way, it moves the research line forward. The paper also demonstrates, if further demonstration is needed, the value of landscape or distributional vs. “site” approaches to the understanding of hunter-gatherer archaeological records. I recommend publication even in the paper’s current form, but also have a few suggestions that might improve it modestly.

Undeniably, the paper bears an Old World Paleolithic (OWP) cast, in its focus upon cores and flakes, implicitly in its focus on cores and retouched flakes rather than formal tools (e.g. points, scrapers, that are more common in the Americas), the central importance of cortex ratio as analytical device, consideration of technologies and “transitional” assemblages and, of course, in its legitimate concern with long-term tool exposure and time frames that are more geological or truly archaeological than shorter time frames. This is understandable, and not a criticism. However, the paper somewhat imperfectly accesses relevant North American literature (i.e. not empirical North American studies but more theoretical work by North Americanists.)

The paper is written fairly well but is remarkably dense in substantive terms, less a criticism than an observation. Certainly it is systematic in defining its problem, designing simulations that can test common assumptions about the meaning of tool and assemblage variation and in reporting of results. Indeed, the latter are given in copious detail that catalogues the complex covariation among variables. I mean this as praise, but it also makes the paper a challenge to read and assimilate. Varying clarity of prose also handicaps the paper’s value. For instance, around lines 875-8, Coco writes, “recycling intensity, which is measured as the ratio of recycled objects to total objects in an assemblage, increases on a landscape scale when there is a higher probability that agents are practicing scavenging behaviors and when agents are more frequently retouching flakes.” Her analysis shows that Coco’s conclusions are not tautological, but this statement invites that misinterpretation. Since scavenging, in this paper’s context, amounts to recycling, and retouch of scavenged/recycled objects amounts to, well, retouch, here she seems to say “recycling [as incidence, proportion or “intensity”] increases when recycling increases and retouch increases when retouch increases.” I recommend rewording here to clarify the paper’s results.

I have reworded/reorganized many of the Results sections to make the takeaways simpler and clearer.

There is also the matter of equifinality, briefly acknowledged around Line 1011. Relationships between tools properties (size, technological origin [i.e. flake vs. nodule/core], incidence of retouch [how many tools among all tools], original vs. discard location) and assemblages (“sites”) and landscapes are quite complex, as the paper shows. Clearly, the paper has important implications (e.g., for the incidence of recycled tools at hotspots qua sites). But so many outcomes contingent upon so many starting conditions will make it difficult to apply Coco’s simulation results to empirical cases. Or rather, it makes the model almost infinitely protean, capable of being used selectively or partially to explain any patterns observed in empirical data. Complexities of equifinality and model specifications are addressed somewhat from Lines 1010-30. She might reflect on and then advise others somewhat more thoroughly about how to address the equifinality challenge in so complex a setting of variables and their interactions.

I have created a table outlining the different expected characteristics of the archaeological record under the main parameters of interest for this paper: recycling frequency, mobility style, and selection behaviors.

One grid-square variable is called “reduction intensity,” by which Coco means the proportion of tools among all tools. This wording is unfortunate, because it confuses recycling incidence (count) and proportion (ratio of recycled tools/all tools) with what many archaeologists, I believe, mean by “intensity” either of reduction or recycling, i.e. how much or to what extent each tool is reduced. This understanding of intensity, of course, relates to the curation concept when it’s defined as the ratio of realized to maximum utility. I strongly urge Coco to reconsider use of “intensity” to mean count or % of retouched tools, and instead to call it recycling “incidence” or “proportion.” These terms are both more accurate and recognize that “intensity” often—usually?—pertains to degree of retouch of individual tools.

I agree with the reviewer that using “intensity” was not ideal given the varied use of that term in archaeology. I have updated the instances of “recycling intensity” to be “recycling incidence” or
“recycling incidence ratio” where appropriate.

On a related matter, one element omitted from the research design is degree or extent of recycling, which amounts to extent of reduction, i.e., what I’d call, contra Coco, reduction intensity. This is understandable because the behavior of so many other variables is modeled, and one more might greatly complicate analysis, interpretation and reporting of results. But extent of curation is important both empirically (notably in Dibble’s original reduction thesis, itself imported to the Americas in Hoffman’s mid-1980s paper) and in theory or concept, where—e.g. in my 1996 Jnl. of Anthr. Res. paper—it is defined as a ratio between realized and maximum utility. Granted, in Coco’s OW Paleolithic context, curation so defined requires further specification owing to possible differences in maximum utility depending upon raw-material supply and technology over geological time. That is, what was “used up” in, say the Middle Pal. might be considered still useful in the Upper Pal. But the modeling described here treats recycling as a categorical variable (i.e. either a tool was recycled or it wasn’t) when the curation that underlies it actually is a continuous one. I don’t know but wonder if categorical “recycling” affects model results. But I suspect as much when results are expressed as “intensity” (again, incidence or proportion) of recycled tools compared to landscape variables. If “intensity” is not mere incidence, but considered as varying amount of reduction per categorically recycled tool, then model results for recycled-tool incidence/proportion will differ from those reported here if degree of reduction—again, curation—varies both significantly and continuously among tools. Two tools can be equally recycled, one of them slightly and the other greatly. Collapsing this variation into “presence of recycling” seems a serious model limitation. I hope that Coco will integrate the continuous nature of tool reduction (i.e., extent of reduction) in future modeling. Somewhat obliquely, this matter is addressed ca. Line 910 where Coco briefly acknowledges the effect of use-life as a limited, not infinite, quantum. But that passage essentially says “I haven’t considered this [yet?].” That’s fine, but she might do more than that, for instance by considering how use-life and the linked (but not identical) process of curation can directly affect her model results.

I understand the reviewer’s concern with modeling recycled artifacts as a presence/absence variable rather than a continuous one. However, given the current difficulties of identifying recycled artifacts in the archaeological record (the only real reliable method being the presence of double patina), I think it is more important to examine presence patterns at the start before trying to incorporate a “degree of recycling” variable that does not currently have a reliable archaeological correlate. Additionally, because recycling refers to a very particular type of reduction, requiring that some period of discard has occurred before another phase of reduction happens, it could end up being rather difficult to truly distinguish a degree of recycling from the overall degree of reduction since it might be impossible to distinguish an actual recycling event from a simple edge maintenance event for example.

I have added a few more sentences to the paragraph discussing the connection between use-life of artifacts and the relationship between recycling and exposure time to address how use-life might affect this relationship. As for the issue of curation, please see the comments below.

“Curation” occurs only once in the text where, like the titles of two cited papers in which it also appears, it passes undefined. The text occurrence might imply some equivalence between curation and “technological efficiency,” also undefined. Coco’s citations 63-68 are merely a selection, of course, and not necessarily the most representative ones, of the curation issue. Indeed, the first of these citations—Bamforth, mid-1980s—is a conceptually unclear paper that, among other things, inspired my efforts to try to clarify both the curation concept and the technological-organization approach that Bamforth misunderstood. This is no mere semantic quibble. Conceptually, recycling is subsumed by curation, which makes the latter directly relevant here. At the same time, Lines 420-30 here caused me to usefully rethink the curation concept in the context of Dibble’s reduction thesis, as a process that unfolds not only—or at times perhaps even mostly—in ecological or real-time but geological or archaeological chronological scales as well.

I don’t think that recycling as it is defined in this paper is necessarily subsumed by curation. Curation in the many ways it is defined implies conceptualization of stone tool use within a single population. Curation is often defined in the context of anticipation of future use, which necessarily implies a single agent having that forethought; a tool user unrelated to the original tool producer cannot really anticipate future use of something they themselves did not create. Furthermore, taking curation as a ratio of realized to maximum utility, this also assumes a single assessment of the utility of a tool, which is likely not shared by all tool users that interact with artifacts on geological time scales. I have added a brief discussion of the difference between these concepts in the introduction.

Accordingly, like the Barton and Riel-Salvatore paper that it cites, this ms. has, from an American perspective, a highly constrained view of assemblage variation, limiting it essentially to variation by the proportion or incidence of retouched/recycled tools vs. nodules and, presumably, debris. Again, that quality reflects its OWP cast, but one useful direction for future research is to define assemblage variation more broadly, both in terms of number of “types,” (sidestepping for the moment the validity or integrity of types, especially as defined in OW Middle Paleolithic context and which, anyway, Dibble’s thesis somewhat reconceptualized as various tool “types” representing progressive stages in the degree and pattern of retouch of original flake blanks/tools) and the size-composition relationships that govern assemblage accumulations (sensu my 2010 paper and Phillips et al.’s recent Asian Perspectives paper; for instance, Lines 234-5 make a similar point more extensively elaborated in mine of 2010). One possibility, as above, reconceives the many Middle Paleolithic Bordean “types,” for instance, as Dibble did: size-shape transformations of varying type and degree via reduction of a few basic technological flake-tool types. In this context as in others (again, my 2010 and Phillipps et al. recent paper), assemblage composition as approximated by proportions of Bordean “types” patterns clearly with assemblage size as a function perhaps of occupation span or intensity (as detailed in my 2003 chapter in Moloney and Shott, that no one ever read). Coco’s paper documents similar correlations between assemblage properties and patterns of land-use that might be more broadly contextualized by considering other approaches cited in this paragraph.

I have added a section on future directions for this model. Here I address the fact that the model does not consider aspects of assemblages such as composition. I also briefly identify why recycling may impact some of the proposed relationships between these assemblage properties and cultural-behavioral phenomenon like occupation span.

Coco’s discussion around Lines 90-100 essentially is what North American scholars call “niche-construction theory” (e.g. in her sources 32 and 66). As a substantive matter, that’s neither here nor there, but Coco might acknowledge this parallel so as to clarify the conceptual similarities in her and their approaches. Continuing, the discussion on Lines 99-110 or so, greatly elaborated upon later, put me in mind of Surovell’s model (in his 2012 book) that related occupation span to the ratio between two quantities: formal tools made of nonlocal cherts to used flakes made of local cherts. Like hers, Surovell’s model is simplified as it must be; simulation always identifies key variables and charts their covariation under various conditions, not recreate the past in all details. But Coco’s model seems to build on, even surpass Surovell’s in its greater complexities of land-use and “site” occupation and in consideration of repeated, if intermittent, use of flakes and/or nodules. Still, citing Surovell’s model better contextualizes Coco’s own work.

I have added citations to indicate the parallel between niche construction theory and the sites as sources comment in the introduction.

Surovell’s model uses ratios of local:non-local materials and debitage:transported tools to make arguments about occupation span of sites. Given that Surovell’s models are dependent on use-life of artifacts and that his own simulations show that large use-life variables lead to relatively little change in the proportions of different classes of artifacts across time, I think that the infinite use-life assumed in my model might preclude any current assessment of how Surovell’s model is related to my own. I have added a reference to Surovell’s work in my discussion about adding in use-life of artifacts as a future direction for the model.

The Discussion section is unusually long and somewhat repetitive. It might be revised certainly by editing and shortening, and perhaps by reorganization as numbered sections that each address one of the various points made in the results section and repeated there.

I have reorganized the Discussion section so it is more streamline and less repetitive. I have added subheadings to the discussion section to drive home the important points of the paper.

The text is clean, with only two copy-editing glitches that I caught:
Line 32: “those that [have] been exposed”
Line 64: “the productions and flakes” [the production of flakes??]

These changes have been made.
